# New Biologically Hybrid Pharmacophore Thiazolidinone-Based Indole Derivatives: Synthesis, In Vitro Alpha-Amylase and Alpha-Glucosidase along with Molecular Docking Investigations

**DOI:** 10.3390/molecules27196564

**Published:** 2022-10-04

**Authors:** Shoaib Khan, Shahid Iqbal, Fazal Rahim, Mazloom Shah, Rafaqat Hussain, Hamad Alrbyawi, Wajid Rehman, Ayed A. Dera, Liaqat Rasheed, H. H. Somaily, Rami Adel Pashameah, Eman Alzahrani, Abd-ElAziem Farouk

**Affiliations:** 1 Department of Chemistry, Hazara University, Mansehra 21120, Pakistan; 2 Department of Chemistry, School of Natural Sciences (SNS), National University of Science and Technology (NUST), H-12, Islamabad 46000, Pakistan; 3 Department of Chemistry, Abbottabad University of Science and Technology (AUST), Abbottabad 22500, Pakistan; 4 Pharmaceutics and Pharmaceutical Technology Department, College of Pharmacy, Taibah University, Medina 42353, Saudi Arabia; 5 Department of Clinical Laboratory Sciences, College of Applied Medical Sciences, King Khalid University, P.O. Box 9004, Abha 61413, Saudi Arabia; 6 Research Center for Advanced Materials Science (RCAMS), King Khalid University, P.O. Box 9004, Abha 61413, Saudi Arabia; 7 Department of Physics, Faculty of Science, King Khalid University, P.O. Box 9004, Abha 61413, Saudi Arabia; 8 Department of Chemistry, Faculty of Applied Science, Umm Al-Qura University, Makkah 24230, Saudi Arabia; 9 Department of Chemistry, College of Science, Taif University, P.O. Box 11099, Taif 21944, Saudi Arabia; 10 Department of Biotechnology, College of Science, Taif University, P.O. Box 11099, Taif 21944, Saudi Arabia

**Keywords:** synthesis, α-amylase, α-glucosidase enzymes, thiazolidinone, indole, SAR, molecular docking

## Abstract

Amylase and glucosidase enzymes are the primary harmful source in the development of the chronic condition known as diabetes mellitus. The main function of these enzymes is to break the macromolecules into simple sugar units which are directly involved in the solubility of blood, hence increasing blood glucose levels. To overcome this effect, there is a need for a potent and effective inhibitor that inhibits the conversion of macromolecules of sugar into its smaller units. In this regard, we synthesized thiazolidinone-based indole derivatives (1–20). The synthesized derivatives were evaluated for α-amylase and α-glucosidase inhibitory activity. Different substituted derivatives were found with moderate to good potentials having IC_50_ values ranging, for α-amylase, from 1.50 ± 0.05 to 29.60 ± 0.40 μM and, for α-glucosidase, from IC_50_ = 2.40 ± 0.10 to 31.50 ± 0.50 μM. Among the varied substituted compounds, the most active analogs **four** (1.80 ± 0.70 and 2.70 ± 0.70), **five** (1.50 ± 0.05 and 2.40 ± 0.10, respectively) of the series showed few folds better inhibitory activity than standard drug acarbose (IC_50_ = 10.20 ± 0.10 and 11.70 ± 0.10 μM, respectively). Moreover, structure–activity relationship (SAR) was established and binding interactions were analyzed for ligands and proteins (α-amylase and α-glucosidase) through a molecular docking study.

## 1. Introduction

Diabetes mellitus is a group of metabolic diseases characterized by hyperglycemia resulting from defects in insulin secretion, insulin action, or both. This metabolic disease is non-communicable involving a high mortality rate and huge healthcare costs. Approximately 387 million people were affected by diabetes mellitus and almost 4.90 million fatalities occurred in the year 2014 [1]. Postprandial hyperglycemia (PPHG), which frequently occurs after digesting a meal, is caused when the blood glucose level remains high. Diabetes mellitus is linked to secondary problems such as neuropathy, retinopathy, and cardiovascular disorders, among others [2]. There are two enzymes, Such as α-amylase, which cleaves glycosidic linkages of α-D-(1,4) of glycogen into oligosaccharide, followed by α-glucosidase which further breaks into glucose [3] The activity of these enzymes may be limited by anti-diabetic drugs that control blood glucose levels in people who consume carbohydrate-rich diets [4]. These drugs are Voglibose, Miglitol, and Acarbose, which are the current inhibitors used for the treatment of diabetic mellitus to control PPHG. All these drugs display greater potential against targeted enzymes among acarbose used to treat both enzymes while the remaining two drugs are only used against α-glucosidase. 

**Figure 1:**
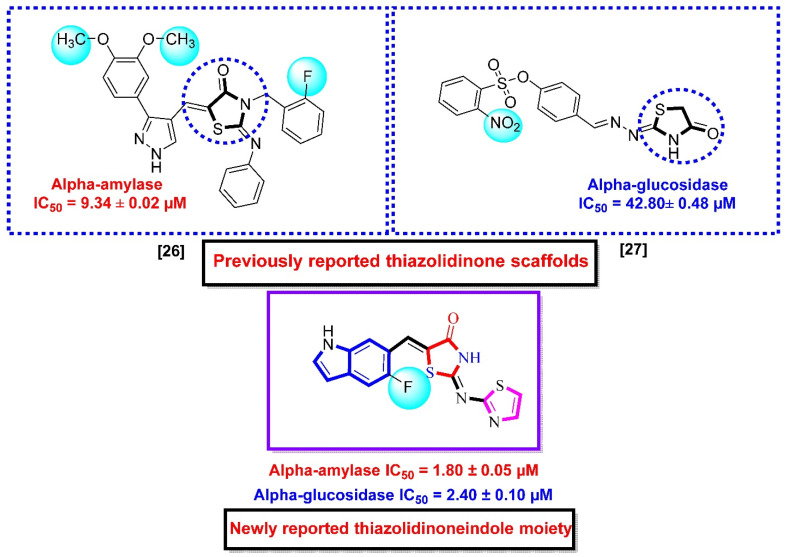
Rationale of the current study.

The inhibitory profile of this drug is not sufficient due to some gastrointestinal side effects [5,6]. The majority of people in the nation are from the lower and middle classes, and he believes that such medications are far more costly. Researchers have tried to extract glucosidase and amylase inhibitors from a variety of sources, including bacteria, fungus, marine algae, and plants [7,8,9,10]. Additionally, a couple of them also researched pure substances, while others studied crude extracts (organic or aqueous) [11,12]. Due to their strong ability to interact with human sugar, glycosidases, and other protein receptors, the iminosugars, a common class of plant and microbial chemicals, have attracted significant study [13,14,15,16]. Most of the pure compounds and extracts were found to be efficient against both these enzymes, but had some complications [17,18]. Thiazolidinone is an adaptable organic molecule with a variety of biological potentials, including anti-bacterial [19], anti-fungal [20], anti-viral [21], anti-inflammatory [22], and anti-tuberculosis [23] effects, similar to other heterocyclic moieties that are utilized as anti-diabetic agents, as well as Alpha-glucosidase and Alpha-amylase inhibitors containing 4-thiazolidinone moiety [24]. The aim of this work is to report the synthetic route for synthesis of thiazolidinone-based indole derivatives and biological significances. These compounds were found with few folds more potent than acarbose against Alpha-amylase and Alpha-glucosidase (enzymes causing diabetes mellitus). Moreover, the results of the present work were compared with previous works, as shown in (Figure 1) [25,26].

## 2. Results and Discussion

### 2.1. Chemistry

**Scheme 1:**
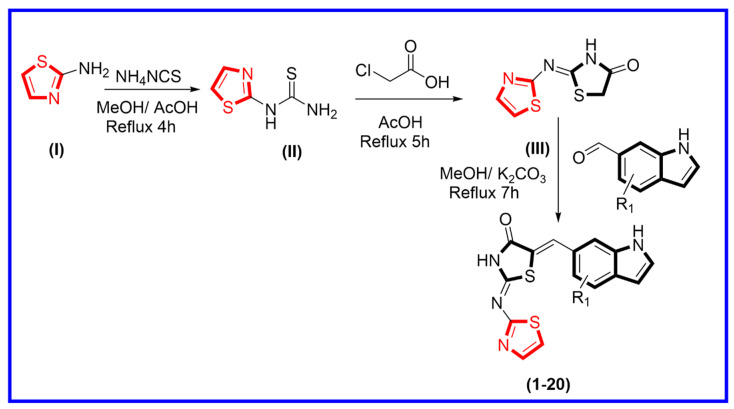
Procedure adopted for the synthesis of thiazolidinone-based indole derivatives (**1–20**).

The synthetic route was adopted for the synthesis of thiazolidinone-based indole derivatives via different stepwise reactions. Initially, 2-aminothiazole (**I**) and ammonium isothiocyanate were mixed and refluxed in the reaction mixture for about 4 h to obtain thiazole-bearing thiourea (**II**). This was further treated with chloroacetic acid in an acidified medium using acetic acid under refluxed condition for about 5 h, affording thiazole-based thiazolidinone moiety (**III**). Equimolar amounts of thiazole-based thiazolidinone (**III**) were further treated with various substituted indole-bearing aldehyde moieties in methanol, and the reaction mixture was then refluxed for about 7 h in the presence of potassium carbonate to produce thiazolidinone-based indole derivatives (**1–20**) (Scheme 1). All reactions were monitored at every step using thin layer chromatography (TLC). Moreover, all the synthesized derivatives were washed with *n*-hexane, in order to remove impurities. Fine powder was collected, and then these products were further characterized through different spectroscopic techniques such as HNMR, CNMR, and HREI-MS. Spectral analysis of the tested analogs is explained in supporting information Section 3.0.

### 2.2. In Vitro Alpha-Amylase and Alpha-Glucosidase Inhibitory Activities

Twenty analogs based on thiazolidinone-based indole (one–twenty) were synthesized and then assessed for their inhibitory potentials against targeted α-amylase and α-glucosidase (in vitro). When evaluated against a standard acarbose drug, all the synthesized thiazolidinone-based indole analogs showed varied ranges on inhibitory potentials ranging from IC_50_ = 1.50 ± 0.05 to 29.60 ± 0.40 μM (against α-amylase) and from IC_50_ = 2.40 ± 0.10 to 31.50 ± 0.50 μM (against α-glucosidase). Structure activity relationship (SAR) studies were carried out for all synthesized thiazolidinone-based indole analogs based on substitution(s) pattern around the indole ring. In order to discuss the SAR studies in a better way, the synthesized analogs were split into thiazole, thiazolidinone, and indole rings. By keeping the thiazole and thiazolidinone rings constant, the variation in inhibitory potentials for both α-amylase and α-glucosidase were observed by changing the nature, position, number(s), and electron donating or electron withdrawing groups around the indole ring (Table 1).

#### Structure Activity Relationship (SAR) Studies for α-Amylase and α-Glucosidase Inhibitory Activity

The nitro group in the *ortho*- and *meta*-positions, among other substituents, on the phenyl ring, which attached to thiazolidinone moiety, as in the case of one, two, and three, has been identified and showed significant results against α-amylase and α-glucosidase, but scaffold one was found one- and three-folds better than two and three, as well as five-folds more active than standard acarbose as reference inhibitor. Among nitro-substituted analogs, scaffold one, having nitro moiety in the *ortho*-position, exhibited excellent potential for both α-amylase and α-glucosidase. Comparison criteria were set for those analogs bearing nitro group at *meta*-position two with a little bit of a decrease in α-amylase and α-glucosidase potential observed, which might be the position of substituents from *ortho*-position to *meta*-position. By this consideration, it was concluded that replacement of the nitro-group at *meta*-position of the phenyl ring decreases the interactive property of the molecule. The activity of analog **three** decreased due to the presence of chlorine moiety, which causes steric hindrance. In this stage, analog **one** showed better potential against α-amylase and α-glucosidase (Table 1).

Analogs **four** and **five** showed higher α-glucosidase and α-amylase performance in comparison to acarbose. This elevated α-glucosidase and α-amylase performance of analogs **four** and **five** might be due to the presence of two flouro groups that may interact better with the active site of the enzyme through hydrogen bonding. Moreover, the position of flouro moieties around the phenyl ring has a significant effect on activity. Thus, the analog **five**-bearing flouro group at *ortho*-location of the phenyl ring showed better inhibitory potentials than analog **four,** also bearing flouro substituent, but analog **five** was found with remarkable activity as compared to analog **four,** as well as much better potential than acarbose (Table 1).

Analogs **six, seven,** and **eight,** bearing the different number of hydroxyl moiety at *ortho-* **(six),** *ortho/meta-* **(seven),** and *ortho*-positions (**eight),** showed strong interaction against α-amylase and α-glucosidase. Two analogs were found with few-folds better activity than acarbose. By this comparative study, the better inhibitory profile was shown by analog **seven** when compared with acarbose drugs. Analog **seven** bearing two -OH groups showed superior inhibitory profile due to strong hydrogen bonding; therefore, it is ranked one in this group, while analog **six** bearing one -OH moiety was found with smaller interactions than analog **seven,** potentially due to the presence of CF_3_**.** However, analog **eight** was found with comparable activity with acarbose; the lower activity might be the presence of methoxy substituent which increases steric hindrance and thus reduces the biological potential (Table 1).

Analogs having either hydroxyl groups or hydroxyl groups along with a -Cl group have emerged as potent inhibitors of both Alpha-amylase and Alpha-glucosidase enzymes. Analog **eleven** bearing *meta*-OH and the *ortho*-Cl group was recognized as the most active analog among the hydroxyl group bearing moieties. The potency of analog **eleven** was reduced by the introduction of one -OH group on the *meta*-position instead of *ortho*-position, as in analog **twelve**, which might cause steric hindrance. This is because of the presence of a -Cl group at the *ortho*-site which decreases the enzymatic activity. The activity of analog **eleven** was further reduced due to the presence of one -Cl group which might reduce the binding interactions, as in analog **thirteen**, and two -Cl in the case of analog **fourteen**, also decreasing the biological profile of the molecule (Table 1).

**Figure 2:**
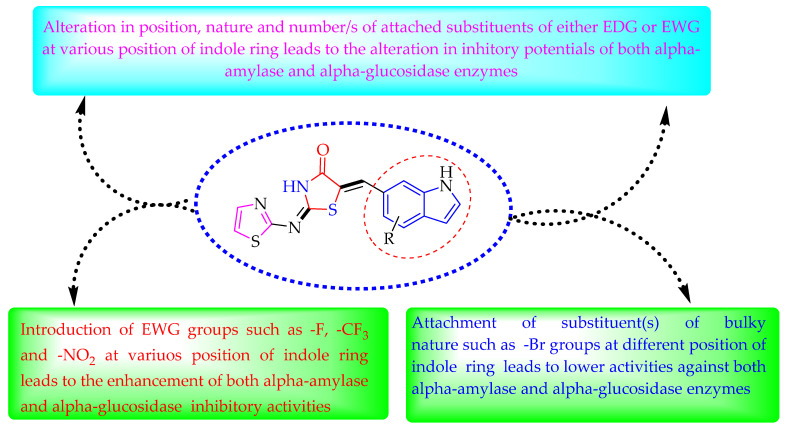
Summary of SAR studies for α-amylase and α-glucosidase inhibitory activities.

Some of the screened analogs were found with lower abilities compared to both α-glucosidase and α-amylase enzymes. When compared with standard drug acarbose (10.20 ±0.10 and 11.70 ± 0.10), these analogs displayed a varied range of inhibitory profile in both the activities, such as analogs **nine** (21.50 ± 0.30 and 23.20 ± 0.40), **ten** (23.20 ± 0.10 and 24.60 ± 0.20), **fifteen** (27.50 ± 0.70 and 28.40 ± 0.80)**, sixteen** (25.20 ± 0.20 and 26.70 ± 0.30)**, seventeen** (29.60 ± 0.40 and 31.50 ± 0.50)**, eighteen** (22.70 ± 0.50 and 23.30 ± 0.40)**, nineteen** (18.40 ± 0.60 and 19.80 ± 0.60), and **twenty** (27.60 ± 0.80 and 29.40 ± 0.90). In the case of hydroxyl groups containing analogs, especially in cases of derivatives **nineteen** and **twenty,** both having a hydroxyl group at the *meta-* and *ortho*-positions, respectively, as well as the bromo group at the *ortho*-position on the same ring. The presence of this bromo moiety and the decrease in activity profile were seen due to its bulky nature which congests the activity profile of the molecule (Figure 2). Thus, both the molecules were found with fewer IC_50_ values (Table 1).

### 2.3. Molecular Docking Study

The molecular docking study investigates the binding mode of interactions with enzyme active sites. Various pieces of software were used for this including auto Dock vina, DSV MGL tool, and Discovery Studio Visualizer [27,28,29,30]. Protein was retrieved from an online source such as a protein data bank (PDB). The designated codes for α-amylase and α-glucosidase enzymes are **1b2y** and **3w37,** respectively.

Molecular docking studies were conducted to explore the binding modalities between ligands and protein. Docking was performed to obtain a population of possible conformations and orientations for the ligand at the binding site. The protein was loaded in auto dock vina software, creating a PDBQT file that contained a protein structure with hydrogen in all polar residues. All bonds of ligands were set to be rotatable. All calculations for protein-fixed ligand-flexible docking were performed using the Lamarckian Genetic Algorithm (LGA) method. The docking site on protein target was defined by establishing a grid box with the dimensions of X: 38.0729, Y: 33.3208, and Z: 25.0000 Å, with a grid spacing of 0.375 Å, centered on X: 20.2892, Y: 10.3219, and Z: 32.3218 Å. The best conformation was chosen with the lowest docked energy after the docking search was completed. Nine runs with AutoDock Vina were performed in all cases per each ligand structure, and for each run the best pose was saved as shown in Table 2 and Table 3. The average affinity for best poses was taken as the final affinity value. The interactions of complex alpha-amylase and -glucosidase protein–ligand conformations, including hydrogen bonds and the bond lengths, were analyzed using DSV.

**Figure 3:**
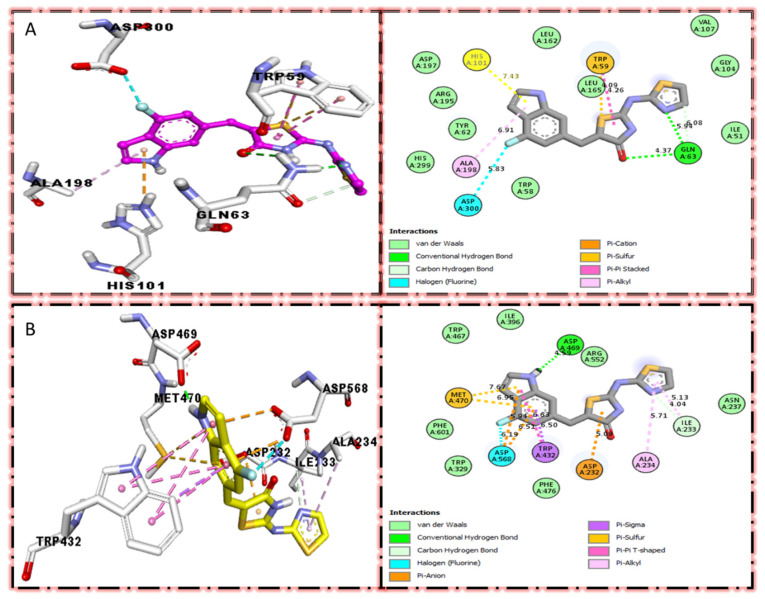
Protein–ligand interaction profile for active analog against the Alpha-amylase and Alpha-glucosidase represents the surface of the corresponding enzyme. (**A**,**B**) indicate the PLI profile for compound 4.

**Figure 4:**
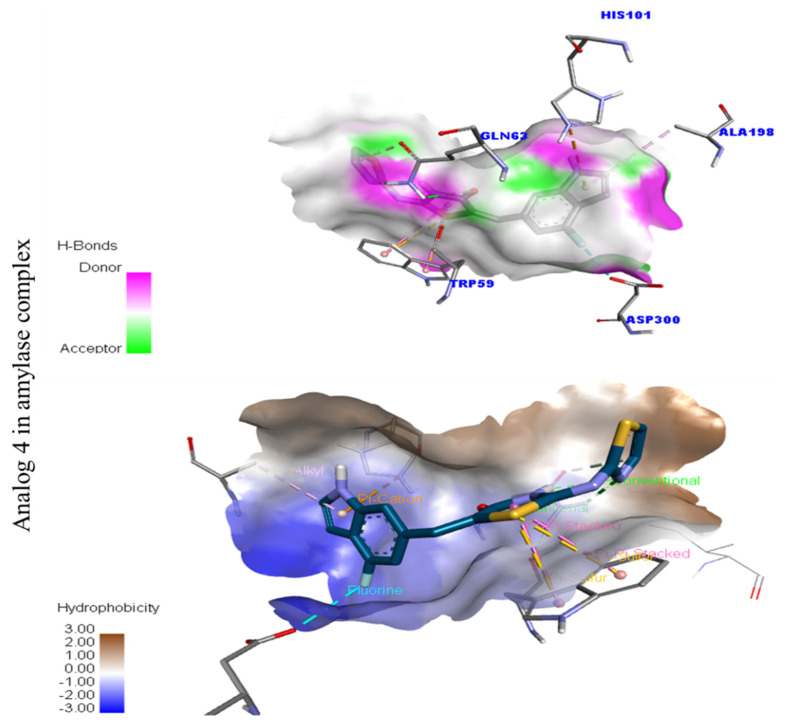
Represent hydrogen bond and hydrophobic character against Alpha-amylase indicate the PLI profile for compound **4**.

**Figure 5:**
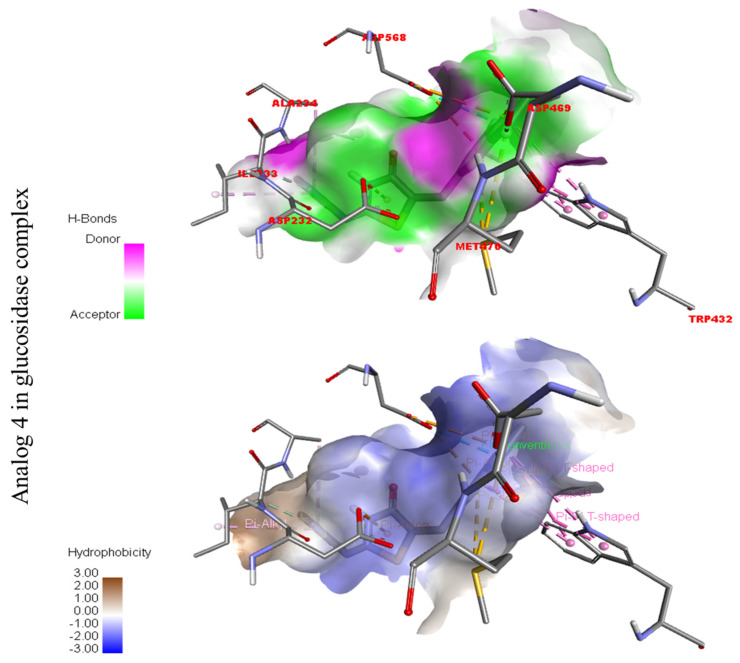
Represent hydrogen bond and hydrophobic character against Alpha-glucosidase indicate the PLI profile for compound **4**.

**Figure 6:**
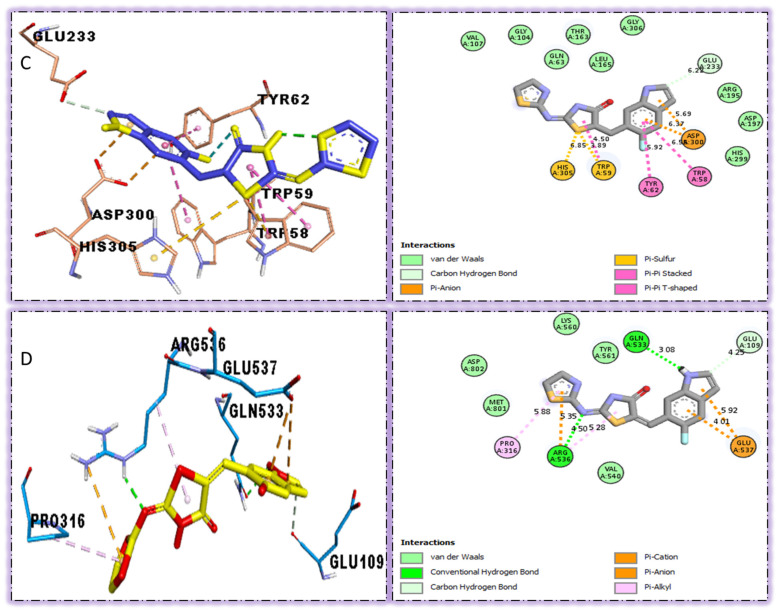
The surfaces of the relevant enzymes are shown by the PLI profiles for powerful drugs against Alpha-amylase and Alpha-glucosidase (**C**,**D**). The PLI profile for chemical **5** is shown by the letters (**C**,**D**).

**Figure 7:**
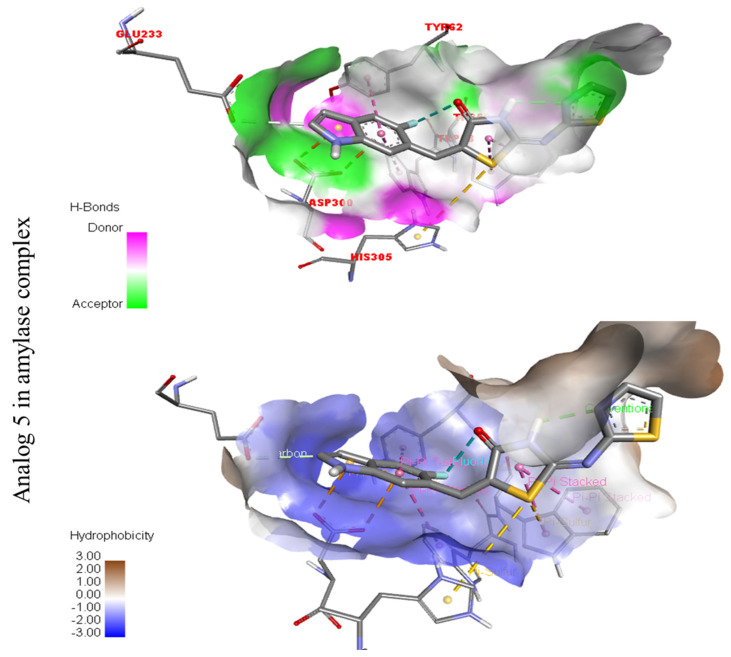
Represent hydrogen bond and hydrophobic character against Alpha-amylase indicate the PLI profile for compound **5**.

**Figure 8:**
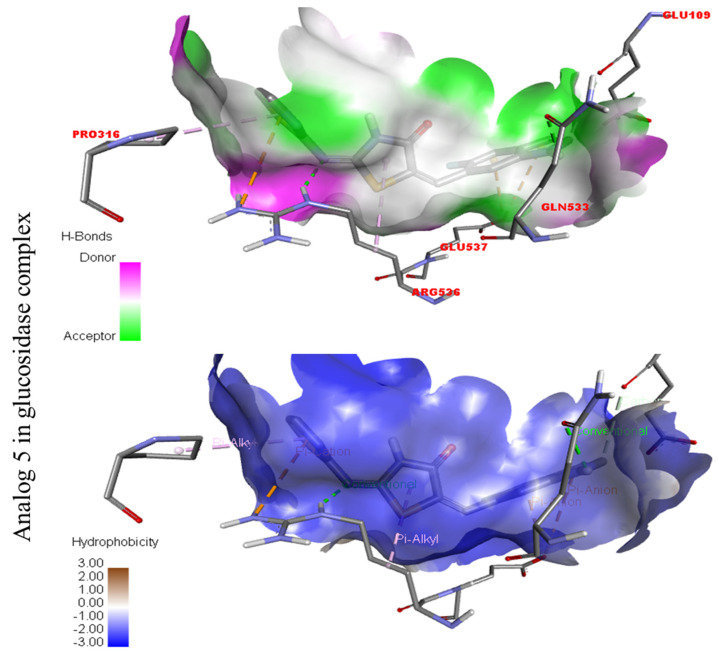
Represent hydrogen bond and hydrophobic character against Alpha-glucosidase indicate the PLI profile for compound **5**.

**Figure 9:**
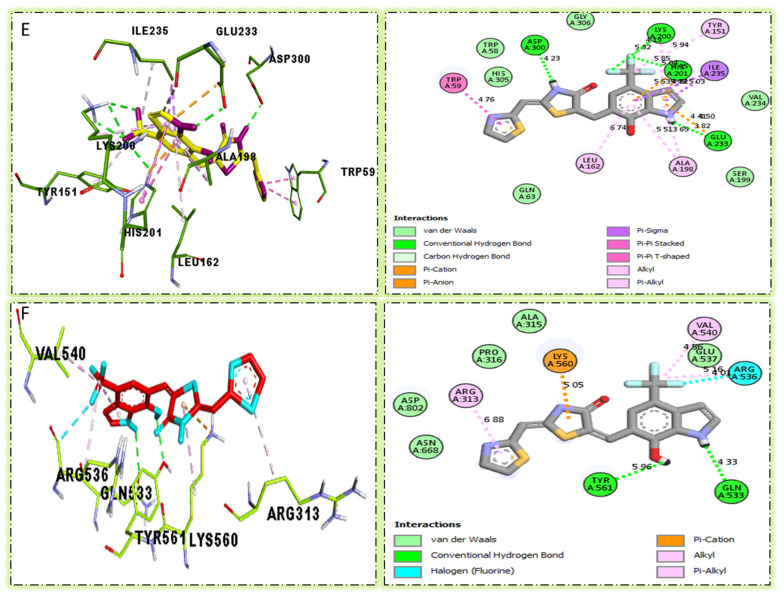
The surface of the appropriate enzyme is shown by the PLI profile for drugs that are effective against Alpha-amylase and Alpha-glucosidase. The PLI profile for chemical **6** is shown by (**E**,**F**).

**Figure 10:**
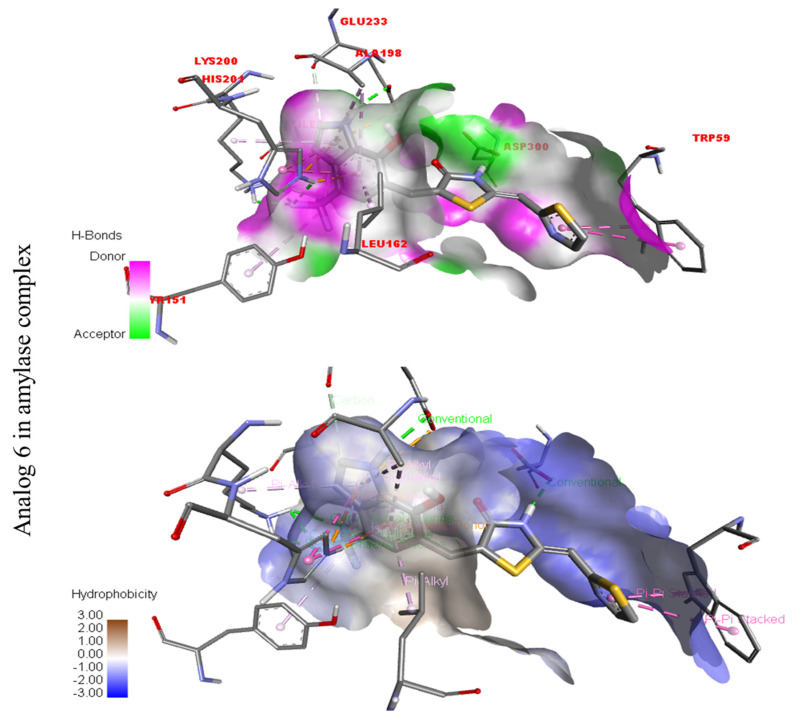
Represent hydrogen bond and hydrophobic character against Alpha-amylase indicate the PLI profile for compound **6**.

**Figure 11:**
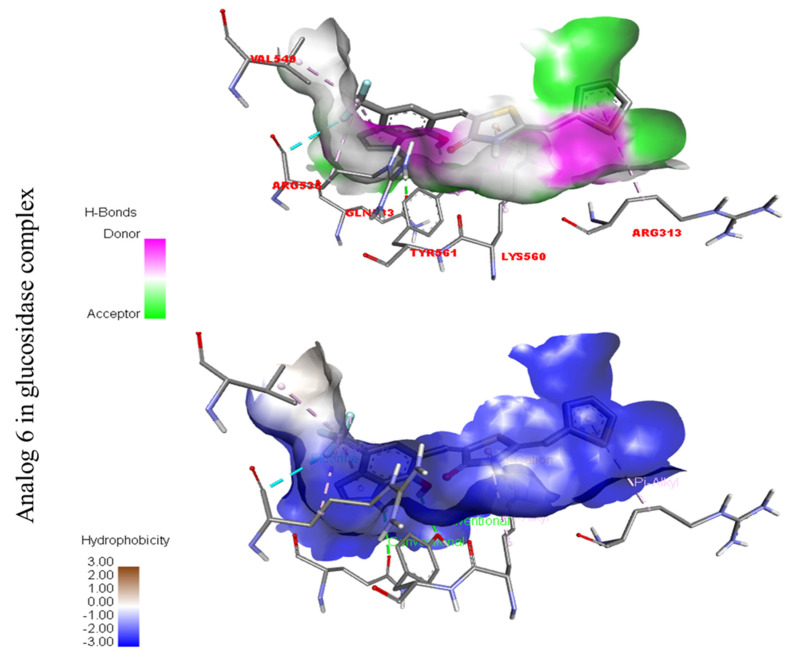
Represent hydrogen bond and hydrophobic character against Alpha-glucosidase indicate the PLI profile for compound **6**.

**Figure 12:**
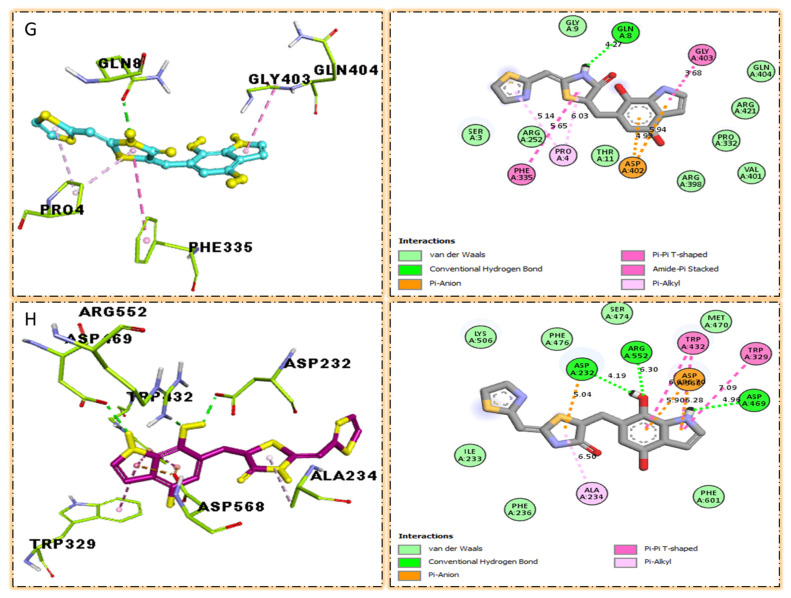
The surface of the relevant enzyme is represented by the PLI profile for drugs that are effective against Alpha-amylase and Alpha-glucosidase. The PLI profile for chemical 7 is shown by (**G**,**H**).

**Figure 13:**
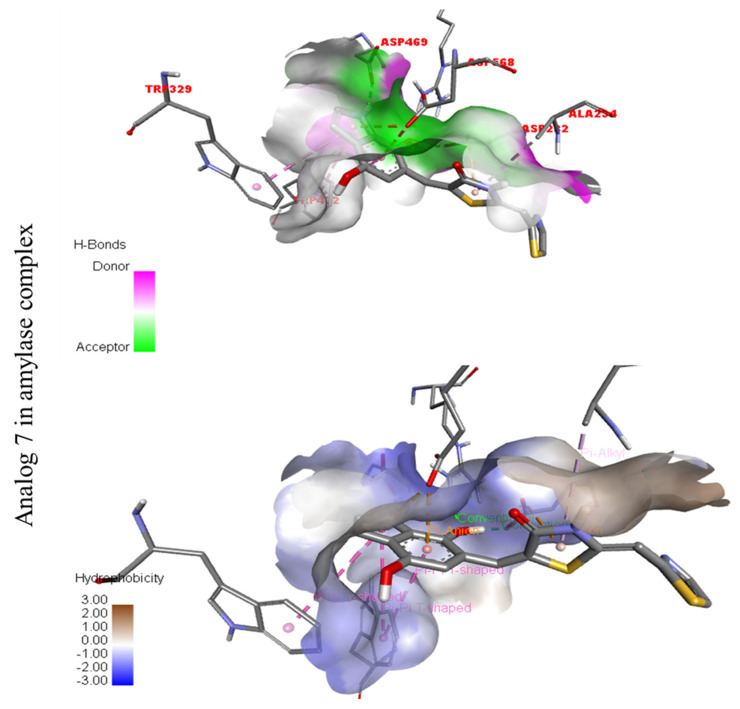
Represent hydrogen bond and hydrophobic character against Alpha-amylase indicate the PLI profile for compound **7**.

**Figure 14:**
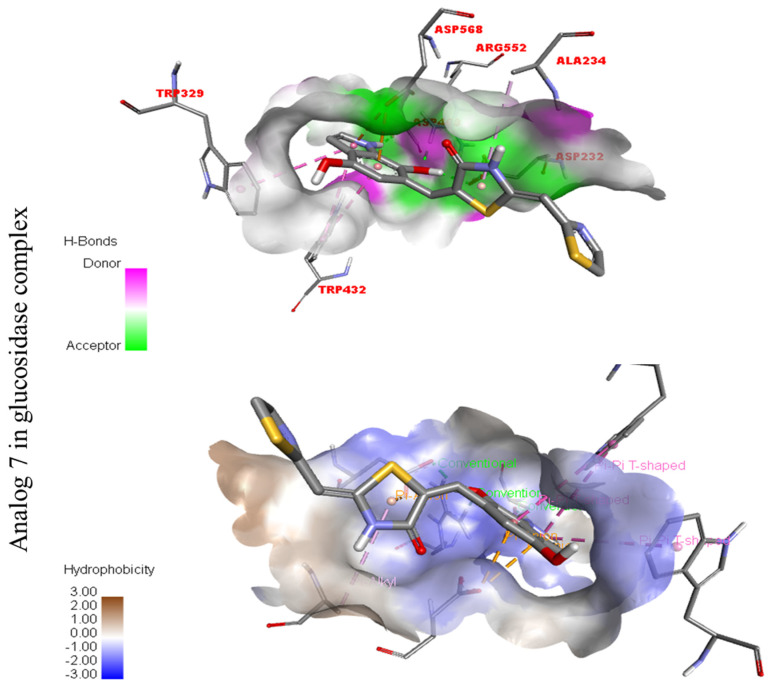
Represent hydrogen bond and hydrophobic character against Alpha-glucosidase indicate the PLI profile for compound **7**.

A molecular docking study revealed the interactive residues with active sites of ligands. The interactions are generally obtained between protein and ligand interest. The target enzymes **Alpha-amylase** and **Alpha-glucosidase** were attained from the online source www.rcsb.org. The potential of ligands against targeted enzymes might be the presence of attached substituents. Here, the nature of substituents is important for binding interactions. Thus, the electron donating group activates the ring by a negative charge, which dominantly increases the interaction toward the positive center or deficient species. In this study, analogs **(four, five, six,** and **seven)** bearing flouro moieties, which are electron withdrawing but have the property to make hydrogen bonds, were found with better interactions, so they were considered as ranked first. They were followed by the interaction procedure of analogs bearing hydroxyl and trifluoro methyl moieties, which also exhibited superposed surface complex structures, as illustrated in (Figure 3, Figure 4, Figure 5, Figure 6, Figure 7, Figure 8, Figure 9, Figure 10, Figure 11, Figure 12, Figure 13 and Figure 14)**.** In addition, these ligands **(four, five, six,** and **seven)** were found with varied binding modalities (Table 2, Table 3, Table 4 and Table 5) against targeted enzymes, which demonstrated excellent potency in vitro study and were found with the best potential (in silico). The best mode of analogs **four** and **five** was observed when compared with the acarbose moiety, and their binding affinity and RMSD value were found lower than that of standard drugs.

In the case of flouro-substituted moiety **four** and its protein–ligand interaction profile exhibited varied interactive residues for Alpha-amylase (A) with distances from 4.09–7.43 A, as shown in (Figure 3). Such residues are HIS-A-101(π-cation), LEU-A-165 (VW), TRP-A-59(π-S), GLN-A-63(H-B), ASP-A-300(H-F), and ALA-A-300(π-R). Similarly, for Alpha-glucosidase (B), the interactive residues, with distances from 4.04–7.67 A, are MET-A-470(π-anion), ASP-A-469(H-B), ILE-A-233(π-X), ALA-A-234(π-R), ASP-A-232(π-anion), TRP-A-432(π-σ), and ASP-A-568(H-F), as shown in (Figure 3).

The strongest molecule in the series, number five, had a fantastic interactions profile with the targeted enzymes. Flouro-substituted analog **five** was found with different interactions against Alpha-amylase (C) with distances from 4.50–6.85 A, such as HIS-A-305(π-S), TRP-A-59(π-S), TYR-A-62(π-π-stacked), TRP-A-58(π-π-T-shaped), and ASP-A-300(π-anion), as shown in (Figure 6). While those against Alpha-glucosidase (D) had distances from 3.08–5.92 A, including Pro-a-316(π-R), ARG-A-536(H-B), GLU-A-537(π-anion), GLU-A-537(π-cation), GLN-A-533(H-B), etc., as shown in (Figure 6).

Compound **six** in the series exhibited a significant PLI profile. Analog **six** containing the CF3-group at the *meta-* and-OH at the *ortho*-positions on the phenyl moiety showed better interactions against Alpha-amylase (E) with distances from 3.82–6.74 A, such as TRP-A-59(π-π-stacked), ASP-A-300(H-B), LYS-A-300(H-B), ASP-A-201(H-B), and TYR-A-151(R), ILE-A-235(π-sigma), GLU-A-233(H-B), ALA-A198(π-R), LEU-A-162(π-R), etc., as shown in (Figure 9). While those against Alpha-glucosidase (F) had distances from 4.33–6.88 A, including ARG-A-313(π-R), LYS-A-560(π-cation), VAL-A-540(R), ARG-A-536(H-F) GLN-A-533(H-B), TYR-A-561(H-B), etc., as shown in (Figure 9).

The third most potent compound **seven** in the series illustrated comparable activity with analogs **four** and **five** having better interaction profiles. Analog **seven** contains nitro moiety which showed different interactions against Alpha-amylase (G) with distance from 3.68–5.94 A, such as PHE-A-335(π-π-stacked), PRO-A-4(π-R), ASP-A-402(π-anion), GLY-A-403(π-π-T-shaped), GLN-A-8(H-B), etc., as shown in (Figure 12). While those against Alpha-glucosidase (H) had distances from 4.19–7.09 A, including ASP-A-232(H-B), ARG-A-552(H-B), TRP-A-432(π-π-T-shaped), TRP-A-329(π-π-T-shaped), ASP-A-469(π-anion), ALA-A-234(π-R), etc., as shown in (Figure 12).

Resultantly, it was observed that analogs **four** and **five** were found to have excellent potential against targeted enzymes. It might be that the presence of electrons withdrawing and donating groups, respectively, increased the binding modalities with different interactions such as pi-anion, pi-cation, pi-sulfur, hydrogen bonding, and pi–pi interaction. The interactive ability of these scaffolds is due to the presence of benzene rings and different heteroatoms.

## 3. Experimental Section

### 3.1. Material and Methods

For the synthesis of desired compounds, all the chemicals and reagents were purchased from the USA (Sigma Aldrich). Thin layer chromatography (TLC) was performed on pre-coated silica gel aluminum plates (Kieselgel 60,254, E. Merck, Germany). Chromatograms were visualized by UV at 254 and 365 nm. High-resolution electron impact mass spectra (HREI-MS) were recorded on a Finnigan MAT-311A (Germany) mass spectrometer. NMR experiments were performed on an Avance Bruker AM 600MHz machine.

### 3.2. General Procedure for the Synthesis of Thiazolidinone-Based Indole Derivatives (**1–20**)

The mixture of 2-aminothiazole (**I,** 1 equivalent) and ammonium isothiocyanate (1 equivalent) was mixed in methanol (10 mL), and the reaction mixture was refluxed in the presence of glacial acetic acid to yield thiazole-based thiourea analog (**II**), which was then treated with chloroacetic acid (1 equivalent) under reflux in acidic medium and afforded thiazole-based thiazolidinone derivatives (**III**). In the next step, compound (**III**, 1 equivalent) was further treated with a different substituted indole-bearing aldehyde (1 equivalent) in methanol (10 mL) and the reaction mixture was refluxed in the presence of potassium carbonate (0.75 equivalents) to yield thiazolidinone-based indole derivatives **(1–20)** in appropriate yield.

## 4. Conclusions

Thiazolidinone-based indole derivatives (**one–twenty**) were synthesized successfully and confirmed through different spectroscopic techniques such as NMR and HR-MS. The biological profile of all the derivatives was screened against α-amylase and α-glucosidase to investigate the better potential of the scaffolds. Among them, few derivatives were found with moderate to good activity, while the most potent behavior was shown in this study in scaffolds **four** (2Z,5E)-5-((4-fluoro-1H-indol-6-yl)methylene)-2-(thiazol-2-ylimino)thiazolidin-4-one(1.80 ± 0.70 and 2.70 ± 0.70) and **five** (2Z,5E)-5-((5-fluoro-1H-indol-6-yl)methylene)-2-(thiazol-2-ylimino)hiazolidine-4-one (1.50 ± 0.05 and 2.40 ± 0.10, respectively) of the series, showing to have few folds better inhibitory activity than standard drug acarbose (IC_50_ = 10.20 ±0.10 and 11.70 ± 0.10 μM, respectively) The interaction of these scaffolds might be due to the presence of flouro moieties forming a strong hydrogen bond with enzyme active sites. The potential of the scaffold depends upon the nature of the substituent, either small in size or bulky group, which increases or decreases the binding interactions. A molecular docking study also revealed the significant binding modalities of subjected candidates. This study identifies a new class of thiazolidinone-based indole derivatives as α-amylase and α-glucosidase inhibitors.

## Figures and Tables

**Table 1:** Substituted thiazolidinone-based indole derivatives along with biological profile (**1–20**).

C.No	R	Alpha-Amylase IC_50_ (µM ± SEM)	Alpha-Glucosidase IC_50_ (µM ± SEM)
**1**	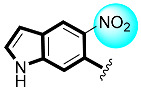	4.70 ± 0.20	5.80 ± 0.20
**2**	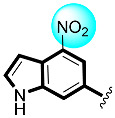	5.60 ± 0.30	6.20 ± 0.30
**3**	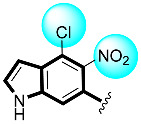	7.90 ± 0.40	8.70 ± 0.20
**4**	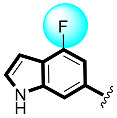	1.80 ± 0.70	2.70 ± 0.70
**5**	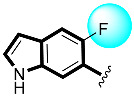	1.50 ± 0.05	2.40 ± 0.10
**6**	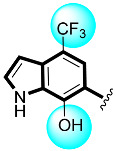	3.20 ± 0.70	4.50 ± 0.70
**7**	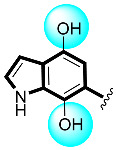	2.60 ± 0.80	3.50 ± 0.80
**8**	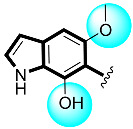	7.60 ± 0.60	8.70 ± 0.80
**9**	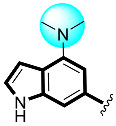	21.50 ± 0.30	23.20 ± 0.40
**10**	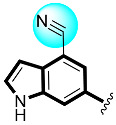	23.20 ± 0.10	24.60 ± 0.20
**11**	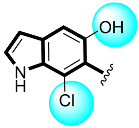	6.90 ± 0.10	7.85 ± 0.20
**12**	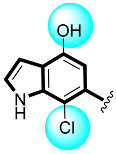	8.30 ± 0.20	9.10 ± 0.30
**13**	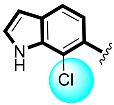	10.70 ± 0.20	11.50 ± 0.20
**14**	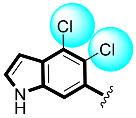	12.20 ± 0.30	13.50 ± 0.40
**15**	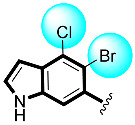	27.50 ± 0.70	28.40 ± 0.80
**16**	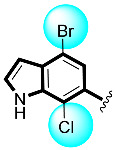	25.20 ± 0.20	26.70 ± 0.30
**17**	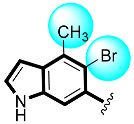	29.60 ± 0.40	31.50 ± 0.50
**18**	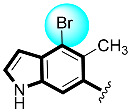	22.70 ± 0.50	23.30 ± 0.40
**19**	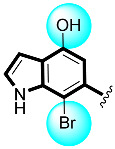	18.40 ± 0.60	19.80 ± 0.60
**20**	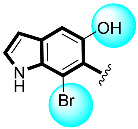	27.60 ± 0.80	29.40 ± 0.90
**Standard drug Acarbose**		**10.20 ± 0.10**	**11.70 ± 0.10**

**Table 2:** Represent the binding affinities and best mode RMSD of analog 4 against Alpha-amylase and Alpha-glucosidase.

Derivative-4 against Alpha-Amylase	**Mode**	**Affinity Kcal/mol**	**Dist. from rmsdl.b**	**Best Mode rmsdu.b**
	**1**	**–8.9**	**0.000**	**0.000**
	**2**	**−8.6**	**3.609**	**9.526**
	**3**	**−8.5**	**3.624**	**9.335**
	**4**	**−7.5**	**2.164**	**2.537**
	**5**	**−7.5**	**3.422**	**4.478**
	**6**	**−7.4**	**2.391**	**3.678**
	**7**	**−7.4**	**3.897**	**9.749**
	**8**	**−7.3**	**3.565**	**4.889**
	**9**	**−7.3**	**4.418**	**6.671**
Derivative-4 against Alpha-Glucosidase	1	−8.3	0.000	0.000
	2	−8.1	4.486	8.557
	3	−6.1	2.593	2.425
	4	−7.8	5.607	8.503
	5	−7.5	4.712	5.490
	6	−7.2	3.462	8.651
	7	−6.9	4.312	8.561
	8	−6.6	4.544	8.481
	9	−6.3	6.601	8.770

**Table 3:** Represent the binding affinities and best mode RMSD of analog 5 against Alpha-amylase and Alpha-glucosidase.

Derivative-5 against Alpha-Amylase	**Mode**	**Affinity Kcal/mol**	**Dist. from rmsdl.b**	**Best Mode rmsdu.b**
	1	−8.7	0.000	0.000
	2	−8.4	2.790	3.819
	3	−8.3	4.664	9.716
	4	−8.3	1.667	2.673
	5	−8.2	3.546	5.504
	6	−8.2	3.440	9.446
	7	−8.1	2.311	8.499
	8	−7.6	12.600	14.644
	9	−7.5	2.996	8.422
Derivative-5 against Alpha-Glucosidase	1	−8.4	0.000	0.000
	2	−8.2	4.674	8.547
	3	−7.9	2.735	3.433
	4	−7.7	6.861	8.414
	5	−7.6	5.548	5.543
	6	−7.5	3.605	4.620
	7	−7.5	6.646	9.757
	8	−7.3	16.481	17.897
	9	−7.1	6.708	9.637

**Table 4:** Represent the binding affinities and best mode RMSD of analog 6 against Alpha-amylase and Alpha-glucosidase.

Derivative-6 against Alpha-Amylase	**Mode**	**Affinity Kcal/mol**	**Dist. from rmsdl.b**	**Best Mode rmsdu.b**
	1	−8.2	0.000	0.000
	2	−7.9	3.424	8.760
	3	−7.8	3.476	8.575
	4	−7.6	4.232	8.661
	5	−7.5	2.704	8.532
	6	−7.5	3.446	4.449
	7	−7.4	3.764	5.355
	8	−7.3	4.575	9.502
	9	−7.3	3.460	3.661
Derivative-6 against Alpha-Glucosidase	1	−7.4	0.000	0.000
	2	−6.7	3.671	4.774
	3	−6.6	5.543	8.623
	4	−6.4	7.883	8.785
	5	−6.1	5.722	8.879
	6	−6.1	6.616	9.405
	7	−6.0	15.492	16.688
	8	−5.9	13.640	18.295
	9	−5.8	6.722	10.103

**Table 5:** Represent the binding affinities and best mode RMSD of analog 7 against Alpha-amylase and Alpha-glucosidase.

Derivative-7 against Alpha-Amylase	**Mode**	**Affinity Kcal/mol**	**Dist. from rmsdl.b**	**Best Mode rmsdu.b**
	1	−7.8	0.000	0.000
	2	−7.7	3.224	8.360
	3	−7.7	3.276	8.575
	4	−7.6	4.132	8.661
	5	−7.4	2.504	8.732
	6	−7.3	3.646	4.549
	7	−7.2	3.764	5.455
	8	−7.2	4.875	9.302
	9	−7.1	3.460	3.561
Derivative-7 against Alpha-Glucosidase	1	−7.3	0.000	0.000
	2	−6.7	3.471	4.674
	3	−6.5	5.543	8.523
	4	−6.4	7.783	8.785
	5	−6.4	5.822	8.379
	6	−6.3	6.316	9.505
	7	−6.0	15.292	16.688
	8	−5.5	13.440	18.295
	9	−5.3	6.822	10.403

## Data Availability

Not applicable.

## Supplementary Materials

The following supporting information can be downloaded at: https://www.mdpi.com/article/10.3390/molecules27196564/s1, Section S3: Spectral analysis. Refs. [31,32] are cited in the supplementary materials.

## Sample Availability

Samples of the compounds are available from the authors.
