# Supplementary material for: New Biologically Hybrid Pharmacophore Thiazolidinone-Based Indole Derivatives: Synthesis, In Vitro Alpha-Amylase and Alpha-Glucosidase along with Molecular Docking Investigations
Source: Molecules. 2022 Oct 4;27(19):6564. doi: 10.3390/molecules27196564 (PMC9571711; doi:10.3390/molecules27196564)
Supplement: Supplementary file 1 [file molecules-27-06564-s001.zip › molecules-1927802-supplementary.pdf]

Supporting Information

New biologically hybrid pharmacophore thiazolidinone-based indole derivatives: Synthesis, in *vitro* α-amylase and α-glucosidase along with molecular docking investigations

Shoaib Khan [1], Shahid Iqbal [2,*], Fazal Rahim [1,*], Mazloom Shah [3], Rafaqat Hussain [1], Hamad Alrbyawi [4], Wajid Rehman [1], Ayed A. Dera [5], Liaqat Rasheed [1], H. H. Somaily [6,7], Rami Adel Pashameah [8], Eman Alzahrani [9] and Abd-ElAziem Farouk [10]

[1] Department of Chemistry, Hazara University, Mansehra 21120, Pakistan
[2] Department of Chemistry, School of Natural Sciences (SNS), National University of Science and Technology (NUST), H-12, Islamabad 46000, Pakistan
[3] Department of Chemistry, Abbottabad University of Science and Technology (AUST), Abbottabad 22500, Pakistan
[4] Pharmaceutics and Pharmaceutical Technology Department, College of Pharmacy, Taibah University,
Medina 42353, Saudi Arabia
[5] Department of Clinical Laboratory Sciences, College of Applied Medical Sciences, King Khalid University, Abha 61413, Saudi Arabia
[6] Research Center for Advanced Materials Science (RCAMS), King Khalid University, P.O. Box 9004,
Abha 61413, Saudi Arabia
[7] Department of Physics, Faculty of Science, King Khalid University, P.O. Box 9004, Abha 61413, Saudi Arabia
[8] Department of Chemistry, Faculty of Applied Science, Umm Al-Qura University, Makkah 24230, Saudi Arabia
[9] Department of Chemistry, College of Science, Taif University, P.O. Box 11099, Taif 21944, Saudi Arabia
[10] Department of Biotechnology College of Science, Taif University, P.O. Box 11099, Taif 21944, Saudi Arabia
* Correspondence: shahidgcs10@yahoo.com (S.I.); fazalstar@gmail.com (F.R.)

3.3. Spectral analysis

***3.3.1. (2Z,5Z)-5-((5-nitro-1H-indol-6-yl)methylene)-2-(thiazol-2-ylimino)thiazolidin-4-one* (1)**

Yield 82%, m.p. 238–40°C, Light yellow. ^{1}H-NMR (600 MHz, DMSO-d_6): δ 11.88 (s, 1H, NH), 11.76 (s, 1H, NH), 8.44 (s, 1H, C-H), 7.56 (d, J = 6.9Hz, 1H, Thiazole-H), 7.35 (s, J = 7.8 Hz, 1H, Thiazole-H), 7.27 (s, 1H, Indole-H), 7.20 (s, 1H, Indole-H), 7.00 (d, J = 7.6Hz, 1H, Indole-H), 6.58 (d, J = 6.1Hz, 1H, Indole-H), ^{13}C-NMR (125 MHz, DMSO-d_6):δ 158.4, 149.5, 136.1, 127.4, 126.6, 125.2, 124.0, 122.5, 121.1, 120.3, 120.2, 118.6, 105.1, 104.2, 100.7. HR EI-MS: *m/z*calcd for $C_{15}H_9N_5O_3S_2$ $[M]^+$ 371.0136; Found: 371.0120.

3.3.2 (2Z,5Z)-5-((4-nitro-1H-indol-6-yl)methylene)-2-(thiazol-2-ylimino)thiazolidin-4-one (2)

Yield 86%, m.p. 236–38°C, Light green. ^{1}H-NMR (600 MHz, DMSO-d_6): δ 11.38 (s, 1H, NH), 11.30 (s, 1H, NH), 8.50 (d, J = 2.3Hz, 1H, Indole-H), 8.48 (d, J = 2.7Hz, 1H, Indole-H), 8.10 (d, J = 7.3Hz, 1H, Indole-H), 7.74 (s, 1H, C-H), 7.70 (d, J = 6.6Hz, 1H, Indole-H), 7.66 (d, J = 7.6Hz, 1H, Thiazole-H), 7.55 (d, J = 8.6Hz, 1H, Thiazole-H), ^{13}C-NMR (125 MHz, DMSO-d_6):δ 168.5, 167.2, 151.5, 148.4, 146.1, 141.9, 141.5, 133.9, 133.4, 122.9, 121.1, 117.9, 115.4, 110.0, 109.4. HR EI-MS: *m/z*calcd for $C_{15}H_9N_5O_3S_2$ $[M]^+$ 371.0134; Found: 371.0118.

3.3.3 (2Z,5Z)-5-((4-chloro-5-nitro-1H-indol-6-yl)methylene)-2-(thiazol-2-ylimino)thiazolidin-4-one (3)

Yield 80%, m.p. 235–37°C, Yellow. ^{1}H-NMR (600 MHz, DMSO-d_6): δ 11.90 (s, 1H, NH), 11.80 (s, 1H, NH), 8.45 (s, 1H, C-H), 7.58 (d, J = 6.7Hz, 1H, Thiazole-H), 7.37 (s, J = 7.9 Hz, 1H, Thiazole-H), 7.29 (s, 1H, Indole-H), 7.01 (d, J = 7.8Hz, 1H, Indole-H), 6.61 (d, J = 6.1Hz, 1H, Indole-H), ^{13}C-NMR (125 MHz, DMSO-d_6):δ 158.6, 149.6, 136.0, 127.6, 126.7, 125.3, 124.1, 122.7, 121.3, 120.4, 120.1, 118.7, 105.2, 104.4, 100.9. HR EI-MS: *m/z*calcd for $C_{15}H_8ClN_5O_3S_2$ $[M]^+$ 404.8606; Found: 404.8510.

3.3.4 (2Z,5Z)-5-((4-fluoro-1H-indol-6-yl)methylene)-2-(thiazol-2-ylimino)thiazolidin-4-one (4)

Yield 81%, m.p. 237–39°C, Light yellow. ^{1}H-NMR (600 MHz, DMSO-d_6): δ 11.87 (s, 1H, NH), 11.78 (s, 1H, NH), 8.79 (d, J = 1.9Hz, 1H, Indole-H), 8.52 (d, J = 2.0Hz, 1H, Indole-H), 7.89 (s, 1H, C-H), 7.57 (d, J = 6.8Hz, 1H, Indole-H), 7.42 (d, J = 8.6Hz, 1H, Indole-H), 7.11 (d, J = 8.2Hz, 1H, Thiazole-H), 7.04 (d, J = 7.5Hz, 1H, Thiazole-H), ^{13}C-NMR (125 MHz, DMSO-d_6):δ 157.7, 131.3, 127.6, 125.5, 122.5, 124.8, 122.6, 121.3, 120.7, 118.6, 118.7, 111.9, 105.1, 104.6, 100.9. HR EI-MS: *m/z*calcd for $C_{15}H_9FN_4OS_2$ $[M]^+$ 344.0228; Found: 344.0212.

3.3.5 (2Z,5Z)-5-((5-fluoro-1H-indol-6-yl)methylene)-2-(thiazol-2-ylimino)thiazolidin-4-one (5)

Yield 92%, m.p. 234–36 °C, Light brown.^{1}H-NMR (600 MHz, DMSO-*d6*): *δ* 11.88 (s, 1H, NH), 11.77 (s, 1H, NH), 8.46 (s, 1H, C-H), 7.59 (d, *J* = 7.8Hz, 1H, Thiazole-H), 7.39 (s, *J* = 7.5Hz, 1H, Thiazole-H), 7.32 (s, 1H, Indole-H), 7.22 (s, 1H, Indole-H), 7.10 (d, *J* = 8.0Hz, 1H, Indole-H), 6.61 (d, *J* = 7.8Hz, 1H, Indole-H), ^{13}C-NMR (125 MHz, DMSO-*d6*):*δ*158.7, 149.6, 136.8, 127.5, 126.4, 125.5, 124.7, 122.9, 121.7, 120.2, 120.5, 118.6, 105.3, 104.4, 100.6. HR EI-MS: *m/z*calcd for $C_{15}H_9FN_4OS_2$ $[M]^+$ 344.0218; Found: 344.0208.

3.3.6 (2Z,5Z)-5-((7-hydroxy-4-(trifluoromethyl)-1H-indol-6-yl)methylene)-2-(thiazol-2-ylimino)thiazolidin-4-one (6)

Yield 87%, m.p. 238–40°C, Yellowish white. ^{1}H-NMR (600 MHz, DMSO-*d6*): *δ* 11.84 (s, 1H, NH), 11.73 (s, 1H, NH), 9.77 (s, 1H, OH), 8.43 (s, 1H, C-H), 7.61 (d, *J* = 7.9Hz, 1H, Thiazole-H), 7.41 (s, *J* = 7.2Hz, 1H, Thiazole-H), 7.33 (s, 1H, Indole-H), 7.13 (d, *J* = 8.2Hz, 1H, Indole-H), 6.63 (d, *J* = 7.1Hz, 1H, Indole-H), ^{13}C-NMR (125 MHz, DMSO-*d6*):*δ* 158.8, 149.7, 146.4, 136.9, 127.6, 126.5, 125.7, 124.9, 122.3, 121.4, 120.1, 120.3, 118.5, 105.1, 104.2, 100.5. HR EI-MS: *m/z*calcd for $C_{16}H_9F_3N_4O_2S_2$ $[M]^+$ 410.0104; Found: 410.0098.

3.3.7 (2Z,5Z)-5-((4,7-dihydroxy-1H-indol-6-yl)methylene)-2-(thiazol-2-ylimino)thiazolidin-4-one (7)

Yield 82%, m.p. 238–41 °C, Light green. ^{1}H-NMR (600 MHz, DMSO-*d6*): *δ* 11.80 (s, 1H, NH), 11.70 (s, 1H, NH), 9.72 (s, 1H, OH), 9.68 (s, 1H, OH), 8.41 (s, 1H, C-H), 7.69 (d, *J* = 8.2Hz, 1H, Thiazole-H), 7.41 (s, *J* = 7.4Hz, 1H, Thiazole-H), 7.31 (s, 1H, Indole-H), 7.19 (d, *J* = 8.0Hz, 1H, Indole-H), 6.61 (d, *J* = 7.6Hz, 1H, Indole-H), ^{13}C-NMR (125 MHz, DMSO-*d6*):*δ* 158.7, 149.5, 136.8, 127.4, 126.3, 125.5, 124.6, 122.1, 121.2, 120.0, 119.9, 118.7, 105.2, 104.3, 100.4. HR EI-MS: *m/z*calcd for $C_{15}H_{10}N_4O_3S_2$ $[M]^+$ 358.0188; Found: 358.0170.

3.3.8 (2Z,5Z)-5-((7-hydroxy-5-methoxy-1H-indol-6-yl)methylene)-2-(thiazol-2-ylimino)thiazolidin-4-one (8)

Yield 91%, m.p. 238–41°C, Light yellow. ^{1}H-NMR (600 MHz, DMSO-d_6): δ 11.89 (s, 1H, NH), 11.83 (s, 1H, NH), 11.03 (s, 1H, OH), 9.69 (s, 1H, C-H), 8.82 (d, J = 7.1Hz, 1H, Thiazole-H), 8.52 (s, J = 6.9Hz, 1H, Thiazole-H), 7.58 (d, J = 6.8Hz, 1H, Indole-H), 7.57 (s, 1H, Indole-H), 6.60 (d, J = 7.2Hz, 1H, Indole-H), 3.18 (s, 3H, CH_3), ^{13}C-NMR (125 MHz, DMSO-d_6):δ 162.0, 161.4, 159.6, 149.6, 146.4, 142.5, 127.7, 127.6, 126.5, 126.2, 125.7, 125.0, 120.7, 118.7, 100.9, 40.3. HR EI-MS: *m/z*calcd for $C_{16}H_{12}N_4O_3S_2$ $[M]^+$ 372.0251; Found: 372.0244.

3.3.9 (2Z,5Z)-5-((4-(dimethylamino)-1H-indol-6-yl)methylene)-2-(thiazol-2-ylimino)thiazolidin-4-one (9)

Yield 93%, m.p. 234–37°C, Light yellow. ^{1}H-NMR (600 MHz, DMSO-d_6): δ 11.90 (s, 1H, NH), 11.78 (s, 1H, NH), 8.81 (s, 1H, C-H), 8.53 (s, 1H, Indole-H), 7.85 (s, 1H, Indole-H), 7.58 (d, J = 6.8Hz, 1H, Thiazole-H), 7.31 (d, J = 7.3Hz, 1H, Thiazole-H), 7.28 (d, J = 9.8Hz, 1H, Indole-H), 6.61 (d, J = 6.2Hz, 1H, Indole-H), 7.01 (d, J=6.9Hz, 1H, Thiazole-H), 2.46 (s, 6H, CH_3), ^{13}C-NMR (125 MHz, DMSO-d_6):δ 158.5, 149.4, 136.1, 127.4, 126.5, 125.2, 124.0, 122.5, 121.4, 120.3, 120.2, 118.6, 105.1, 104.3, 100.8, 45.8, 45.8. HR EI-MS: *m/z*calcd for $C_{17}H_{15}N_5OS_2$ $[M]^+$ 369.0610; Found: 369.0598.

3.3.10 6-((Z)-((Z)-4-oxo-2-(thiazol-2-ylimino)thiazolidin-5-ylidene)methyl)-1H-indole-4-carbonitrile (10)

Yield 82%, m.p. 239–42°C, Light green. ^{1}H-NMR (600 MHz, DMSO-d_6): δ 12.07 (s, 1H, NH), 11.34 (s, 1H, NH), 8.81 (s, 1H, Indole-H), 8.65 (s, 1H, C-H), 8.54 (s, 1H, Indole-H), 7.59 (d, J = 6.6Hz, 1H, Thiazole-H), 7.53 (d, J = 7.7Hz, 1H, Thiazole-H), 7.30 (d, J = 7.6Hz, 1H, Indole-H), 6.62 (d, J = 6.1Hz, 1H, Indole-H), ^{13}C-NMR (125 MHz, DMSO-d_6):δ 151.5, 149.5, 148.2, 145.5,

141.2, 137.6, 128.3, 127.7, 127.5, 121.8, 121.6, 118.6, 111.8, 100.8, 48.5, 39.7. HR EI-MS: *m/z*calcd for $C_{16}H_9N_5OS_2$ $[M]^+$ 351.0130; Found: 351.0125.

3.3.11 (2Z,5Z)-5-((7-chloro-5-hydroxy-1H-indol-6-yl)methylene)-2-(thiazol-2-ylimino)thiazolidin-4-one (11)

Yield 90%, m.p. 236–39°C, Light yellow. ^{1}H-NMR (600 MHz, DMSO-d_6): δ 11.83 (s, 1H, NH), 11.74 (s, 1H, NH), 9.76 (s, 1H, OH), 8.40 (s, 1H, C-H), 7.67 (d, J = 7.2Hz, 1H, Thiazole-H), 7.40 (s, J = 7.3Hz, 1H, Thiazole-H), 7.32 (s, 1H, Indole-H), 7.19 (d, J = 8.0Hz, 1H, Indole-H), 6.60 (d, J = 7.3Hz, 1H, Indole-H), ^{13}C-NMR (125 MHz, DMSO-d_6):δ 158.4, 149.5, 136.5, 127.2, 126.1, 125.4, 124.7, 122.0, 121.0, 120.6, 119.7, 118.5, 105.1, 104.7, 100.6. HR EI-MS: *m/z*calcd for $C_{15}H_9ClN_4O_2S_2$ $[M]^+$ 375.8840; Found: 375.8829.

3.3.12 (2Z,5Z)-5-((7-chloro-4-hydroxy-1H-indol-6-yl)methylene)-2-(thiazol-2-ylimino)thiazolidin-4-one (12)

Yield 83%, m.p. 236–39°C, Yellowish white. ^{1}H-NMR (600 MHz, DMSO-d_6): δ 11.81 (s, 1H, NH), 11.73 (s, 1H, NH), 9.75 (s, 1H, OH), 8.39 (s, 1H, C-H), 7.68 (d, J = 7.4Hz, 1H, Thiazole-H), 7.49 (s, J = 7.7Hz, 1H, Thiazole-H), 7.31 (s, 1H, Indole-H), 7.17 (d, J = 8.2Hz, 1H, Indole-H), 6.68 (d, J = 7.4Hz, 1H, Indole-H), ^{13}C-NMR (125 MHz, DMSO-d_6):δ 158.1, 149.9, 136.5, 127.0, 126.4, 125.3, 124.5, 122.2, 121.8, 120.9, 119.6, 118.4, 105.3, 104.7, 100.1. HR EI-MS: *m/z*calcd for $C_{15}H_9ClN_4O_2S_2$ $[M]^+$ 375.8949; Found: 375.8936.

3.3.13 (2Z,5Z)-5-((7-chloro-1H-indol-6-yl)methylene)-2-(thiazol-2-ylimino)thiazolidin-4-one (13)

Yield 89%, m.p. 235–37°C, Light yellow. ^{1}H-NMR (600 MHz, DMSO-d_6): δ 12.09 (s, 1H, NH), 11.93 (s, 1H, NH), 8.82 (d, J = 7.6Hz, 1H, Thiazole-H), 8.53 (d, J = 6.8Hz, 1H, Thiazole-H), 8.15 (s, 1H, C-H), 8.30 (d, J = 7.3Hz, 1H, Indole-H), 7.90 (d, J = 8.7Hz, 1H, Indole-H), 7.59 (d,

J = 7.4Hz, 1H, Indole-H), 6.62 (d, *J* = 7.2Hz, 1H, Indole-H), ^{13}C-NMR (125 MHz, DMSO-*d*$_6$):*δ* 149.7, 147.8, 140.7, 127.8, 127.7, 120.9, 118.7, 117.6, 116.6, 114.5, 105.3, 104.3, 101.0. HR EI-MS: *m/z*calcd for $C_{15}H_9ClN_4OS_2$ $[M]^+$ 359.9810; Found: 359.9700.

3.3.14 (2Z,5Z)-5-((4,5-dichloro-1H-indol-6-yl)methylene)-2-(thiazol-2-ylimino)thiazolidin-4-one (14)

Yield 91%, m.p. 231–33 °C, Light yellow.^{1}H-NMR (600 MHz, DMSO-*d*$_6$): *δ* 11.79 (s, 1H, NH), 11.68 (s, 1H, NH), 8.32 (s, 1H, C-H), 7.61 (d, *J* = 7.3Hz, 1H, Thiazole-H), 7.33 (s, *J* = 7.6Hz, 1H, Thiazole-H), 7.30 (s, 1H, Indole-H), 7.14 (d, *J* = 8.0Hz, 1H, Indole-H), 6.52 (d, *J* = 7.1Hz, 1H, Indole-H), ^{13}C-NMR (125 MHz, DMSO-*d*$_6$):*δ* 158.0, 149.1, 136.6, 127.3, 126.7, 125.8, 124.9, 122.3, 121.1, 120.5, 119.4, 118.5, 105.3, 104.3, 100.1. HR EI-MS: *m/z*calcd for $C_{15}H_8Cl_2N_4OS_2$ $[M]^+$ 393.8210; Found: 393.8198.

3.3.15 (2Z,5Z)-5-((5-bromo-4-chloro-1H-indol-6-yl)methylene)-2-(thiazol-2-ylimino)thiazolidin-4-one (15)

Yield 88%, m.p. 241–43°C, Light brown. ^{1}H-NMR (600 MHz, DMSO-*d*$_6$): *δ* 11.78 (s, 1H, NH), 11.67 (s, 1H, NH), 8.31 (s, 1H, C-H), 7.59 (d, *J* = 7.8Hz, 1H, Thiazole-H), 7.31 (s, *J* = 7.6Hz, 1H, Thiazole-H), 7.28 (s, 1H, Indole-H), 7.11 (d, *J* = 7.2Hz, 1H, Indole-H), 6.51 (d, *J* = 7.4Hz, 1H, Indole-H), ^{13}C-NMR (125 MHz, DMSO-*d*$_6$):*δ* 158.0, 149.2, 136.5, 127.2, 126.6, 125.7, 124.8, 122.2, 121.0, 120.4, 119.3, 118.4, 105.2, 104.2, 100.0. HR EI-MS: *m/z*calcd for $C_{15}H_8BrClN_4OS_2$ $[M]^+$ 437.8920; Found: 437.8898.

3.3.16 (2Z,5Z)-5-((4-bromo-7-chloro-1H-indol-6-yl)methylene)-2-(thiazol-2-ylimino)thiazolidin-4-one (16)

Yield 81%, m.p. 240–42°C, Light brown. ^{1}H-NMR (600 MHz, DMSO-*d*$_6$): *δ* 11.82 (s, 1H, NH), 11.62 (s, 1H, NH), 9.39 (s, 1H, OH), 8.36 (s, 1H, C-H), 7.57 (d, *J* = 6.7Hz, 1H, Thiazole-H), 7.33 (s, 1H, Indole-H), 7.10 (d, *J* = 8.1Hz, 1H, Thiazole-H), 6.86 (d, *J* = 8.0Hz, 1H, Indole-H),

6.60 (d, J = 6.1Hz, 1H, Indole-H), ^{13}C-NMR (125 MHz, DMSO-d_6):δ 158.6, 149.6, 145.6, 144.3, 143.1, 142.9, 141.7, 140.8, 136.0, 127.6, 121.3, 118.7, 105.2, 104.4, 100.9. HR EI-MS: m/zcalcd for $C_{15}H_8BrClN_4OS_2$ $[M]^+$ 437.9020; Found: 437.8812.

3.3.17 (2Z,5Z)-5-((5-bromo-4-methyl-1H-indol-6-yl)methylene)-2-(thiazol-2-ylimino)thiazolidin-4-one (17)

Yield 73%, m.p. 242–45°C, Light brown. ^{1}H-NMR (600 MHz, DMSO-d_6): δ 11.76 (s, 1H, NH), 11.66 (s, 1H, NH), 8.32 (s, 1H, C-H), 7.56 (d, J = 7.7Hz, 1H, Thiazole-H), 7.30 (s, J = 7.6Hz, 1H, Thiazole-H), 7.26 (s, 1H, Indole-H), 7.09 (d, J = 7.2Hz, 1H, Indole-H), 6.49 (d, J = 7.3Hz, 1H, Indole-H), ^{13}C-NMR (125 MHz, DMSO-d_6):δ 158.3, 149.2, 136.4, 127.2, 126.5, 125.7, 124.6, 122.9, 121.5, 120.7, 119.1, 118.4, 105.2, 104.2, 100.0, 42.5. HR EI-MS: m/zcalcd for $C_{16}H_{11}BrN_4OS_2$ $[M]^+$ 497.9120; Found: 497.9108.

3.3.18 (2Z,5Z)-5-((4-bromo-5-methyl-1H-indol-6-yl)methylene)-2-(thiazol-2-ylimino)thiazolidin-4-one (18)

Yield 79%, m.p. 239–42°C, Light brown. ^{1}H-NMR (600 MHz, DMSO-d_6): δ 11.74 (s, 1H, NH), 11.65 (s, 1H, NH), 8.31 (s, 1H, C-H), 7.55 (d, J = 7.6Hz, 1H, Thiazole-H), 7.27 (s, J = 7.6Hz, 1H, Thiazole-H), 7.25 (s, 1H, Indole-H), 7.08 (d, J = 7.3Hz, 1H, Indole-H), 6.46 (d, J = 7.8Hz, 1H, Indole-H), ^{13}C-NMR (125 MHz, DMSO-d_6):δ158.4, 149.1, 136.3, 127.1, 126.6, 125.8, 124.9, 122.7, 121.4, 120.6, 119.2, 118.3, 105.1, 104.7, 100.3, 42.4. HR EI-MS: m/zcalcd for $C_{16}H_{11}BrN_4OS_2$ $[M]^+$ 497.9108; Found: 497.9096.

3.3.19 (Z)-5-((Z)-3-hydroxybenzylidene)-2-(thiazol-2-ylimino)thiazolidin-4-one (19)

Yield 74%, m.p. 237–38°C, Light yellow. ^{1}H-NMR (600 MHz, DMSO-d_6): δ 11.83 (s, 1H, NH), 11.76 (s, 1H, NH), 9.74 (s, 1H, OH), 8.38 (s, 1H, C-H), 7.66 (d, J = 7.4Hz, 1H, Thiazole-H), 7.45 (s, J = 7.7Hz, 1H, Thiazole-H), 7.29 (s, 1H, Indole-H), 7.15 (d, J = 8.2Hz, 1H, Indole-H), 6.65 (d, J = 7.4Hz, 1H, Indole-H), ^{13}C-NMR (125 MHz, DMSO-d_6):δ 158.3, 149.7, 136.4, 127.1, 126.3, 125.2, 124.4, 122.3, 121.7, 120.7, 119.5, 118.3, 105.2, 104.8, 100.3. HR EI-MS: m/zcalcd for $C_{15}H_9BrN_4O_2S_2$ $[M]^+$ 491.1940; Found: 491.1922.

3.3.20 (2Z,5Z)-5-((7-bromo-5-hydroxy-1H-indol-6-yl)methylene)-2-(thiazol-2-ylimino)thiazolidin-4-one (20)

Yield 76%, m.p. 234–37°C, Light brown. ^{1}H-NMR (600 MHz, DMSO-d_6): δ 11.81 (s, 1H, NH), 11.72 (s, 1H, NH), 9.74 (s, 1H, OH), 8.41 (s, 1H, C-H), 7.65 (d, J = 7.5Hz, 1H, Thiazole-H), 7.43 (s, J = 7.8Hz, 1H, Thiazole-H), 7.35 (s, 1H, Indole-H), 7.18 (d, J = 8.1Hz, 1H, Indole-H), 6.62 (d, J = 7.4Hz, 1H, Indole-H), ^{13}C-NMR (125 MHz, DMSO-d_6):δ 158.3, 149.4, 136.3, 127.1, 126.0, 125.3, 124.6, 122.2, 121.8, 120.5, 119.7, 118.4, 105.0, 104.8, 100.5. HR EI-MS: *m/z*calcd for $C_{15}H_9BrN_4O_2S_2$ [M]$^+$ 491.1928; Found: 491.1912.

Spectral analysis confirms the skeleton of the analogues due to their same basic skeleton different substituents were identified by characterization techniques such as ^{1}HNMR and ^{13}CNMR. The representative spectrum's of compounds as shown in Figure-1–13.

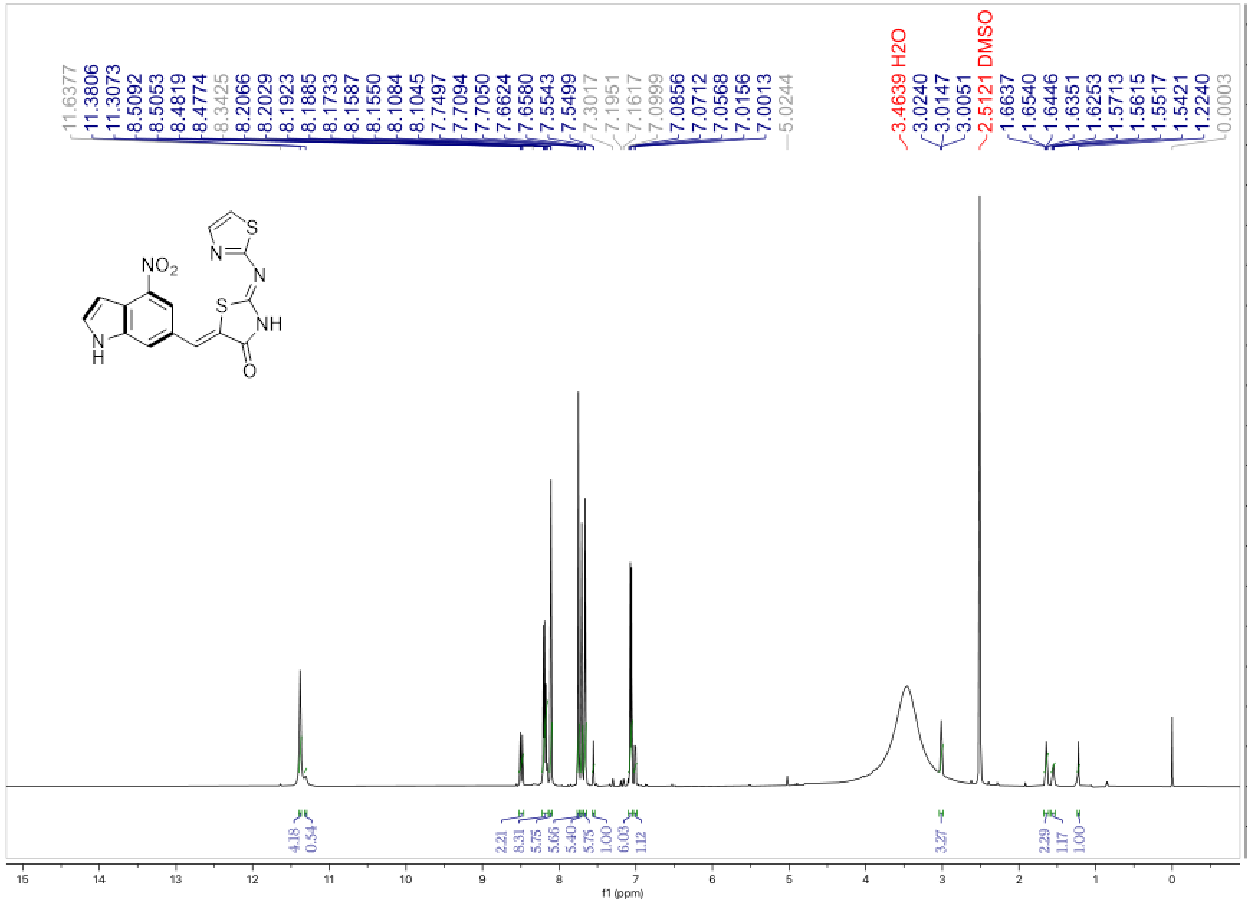

Figure S1. The proton NMR spectrum of (2Z,5Z)-5-((4-nitro-1H-indol-6-yl)methylene)-2-(thiazol-2-ylimino)thiazolidin-4-one (2)

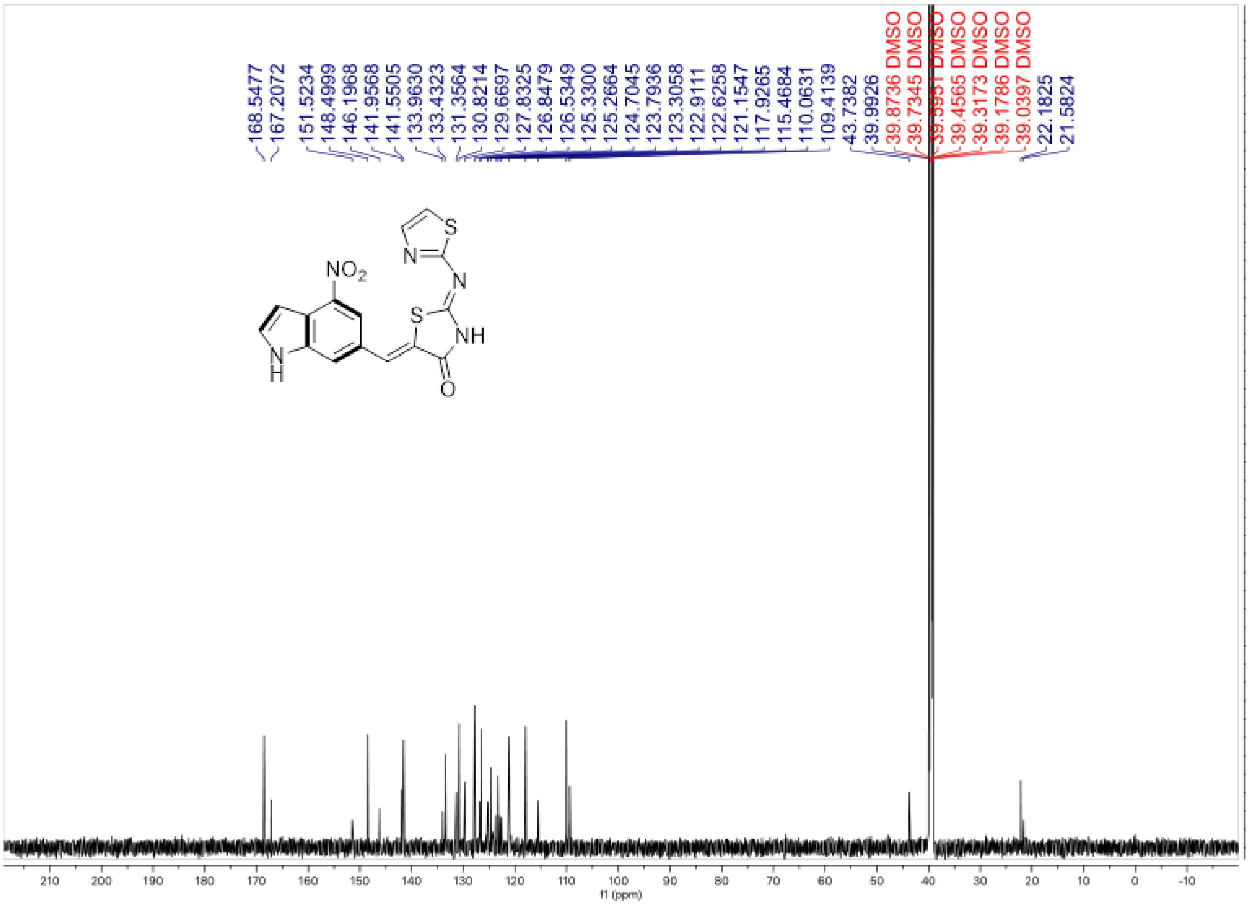

Figure S2. ^{13}C-NMR spectrum of (2Z,5Z)-5-((4-nitro-1H-indol-6-yl)methylene)-2-(thiazol-2-ylimino)thiazolidin-4-one (2)

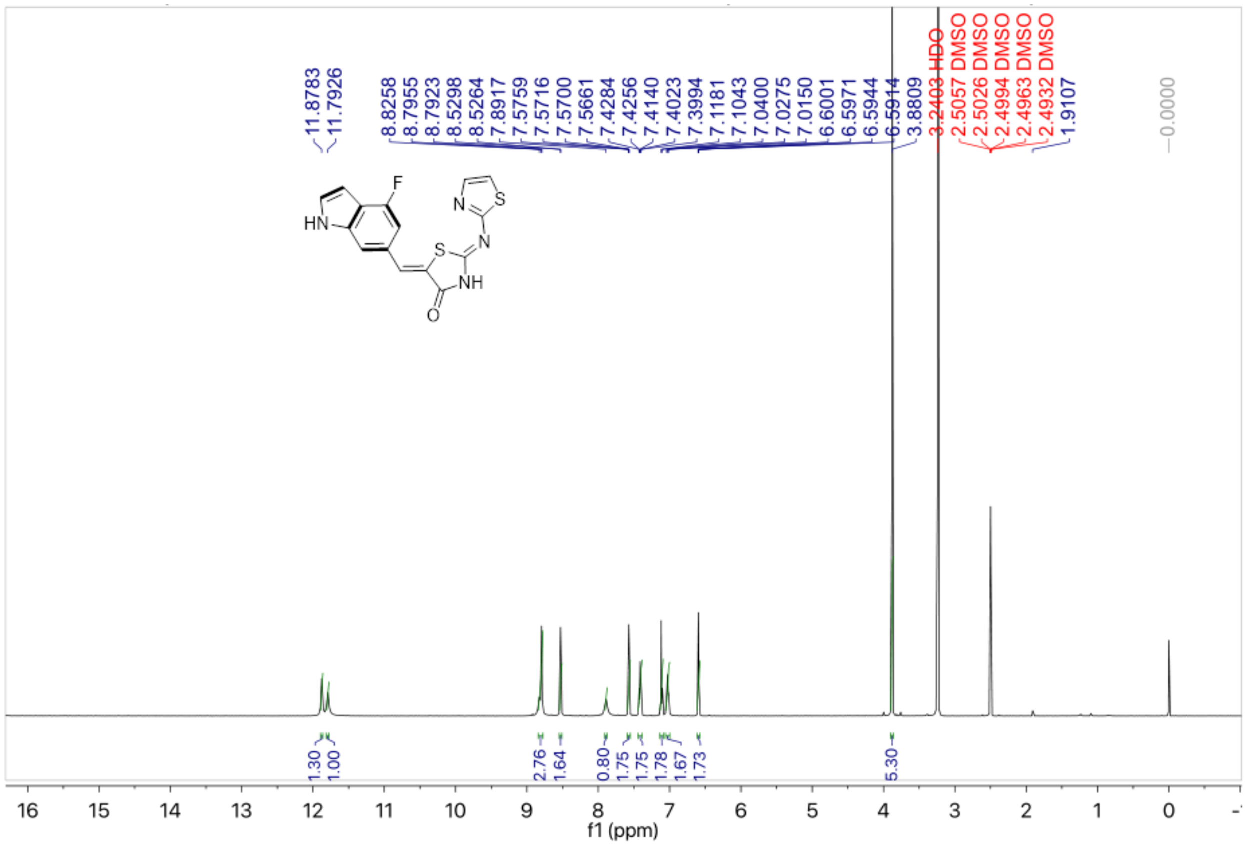

Figure S3. The proton NMR spectrum of (2Z,5Z)-5-((4-fluoro-1H-indol-6-yl)methylene)-2-(thiazol-2-ylimino)thiazolidin-4-one (4)

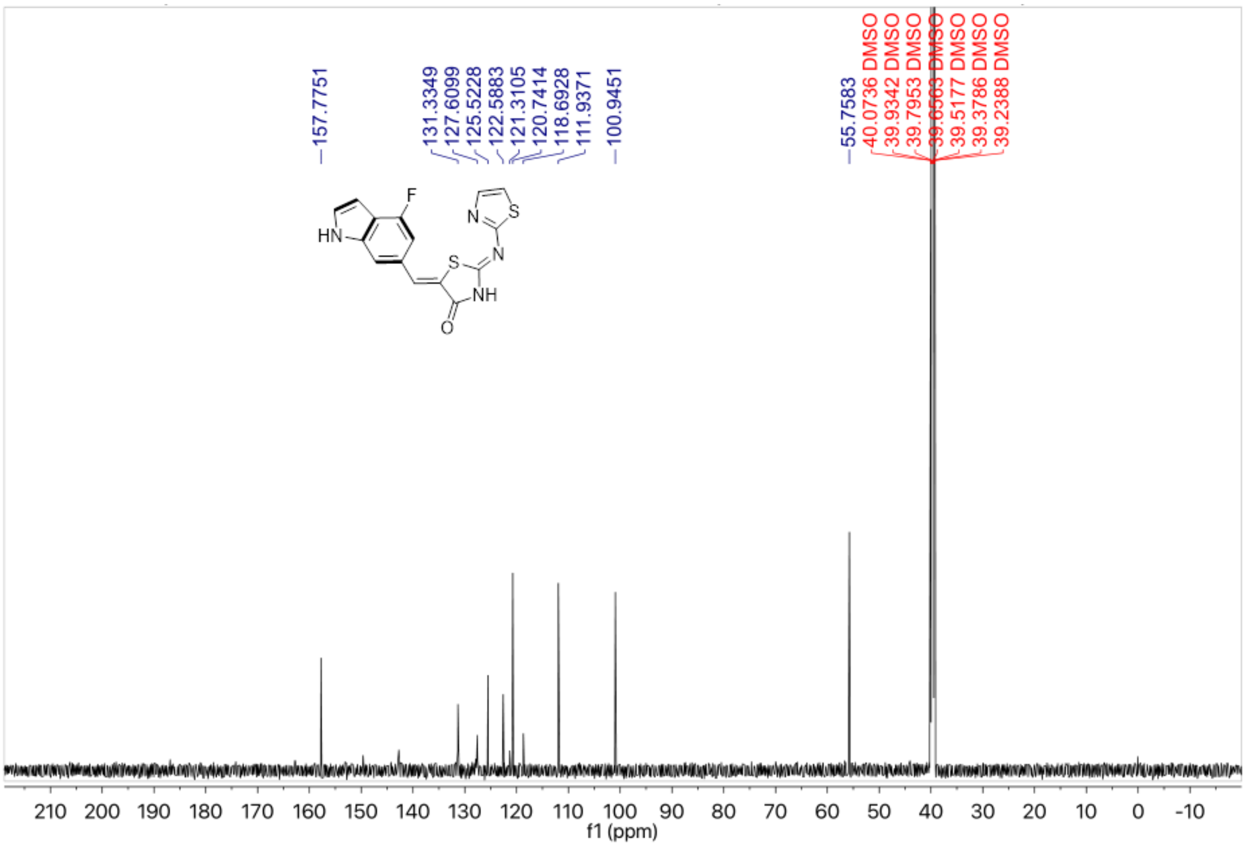

Figure S4. ^{13}C-NMR spectrum of (2Z,5Z)-5-((4-fluoro-1H-indol-6-yl)methylene)-2-(thiazol-2-ylimino)thiazolidin-4-one (4)

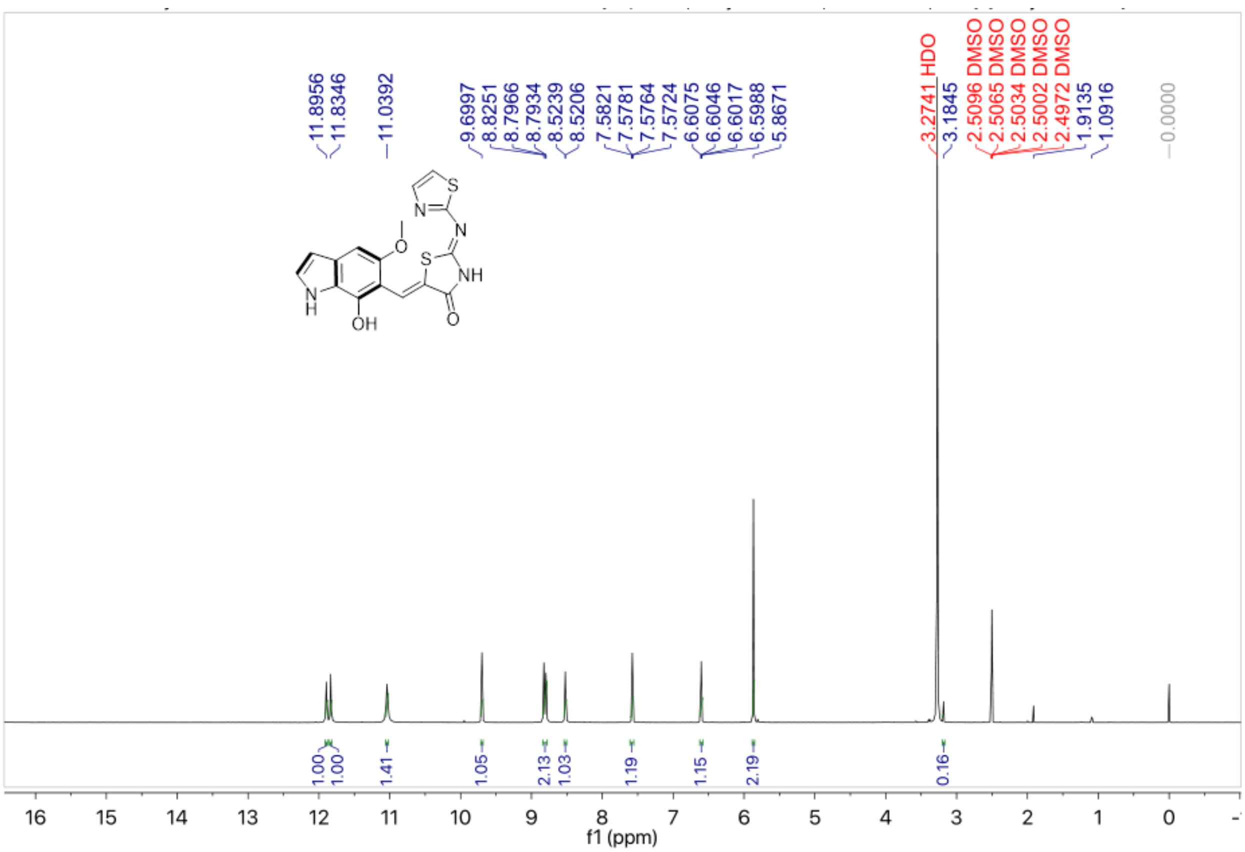

Figure S5. The proton NMR spectrum of (2Z,5Z)-5-((7-hydroxy-5-methoxy-1H-indol-6-yl)methylene)-2-(thiazol-2-ylimino)thiazolidin-4-one (8)

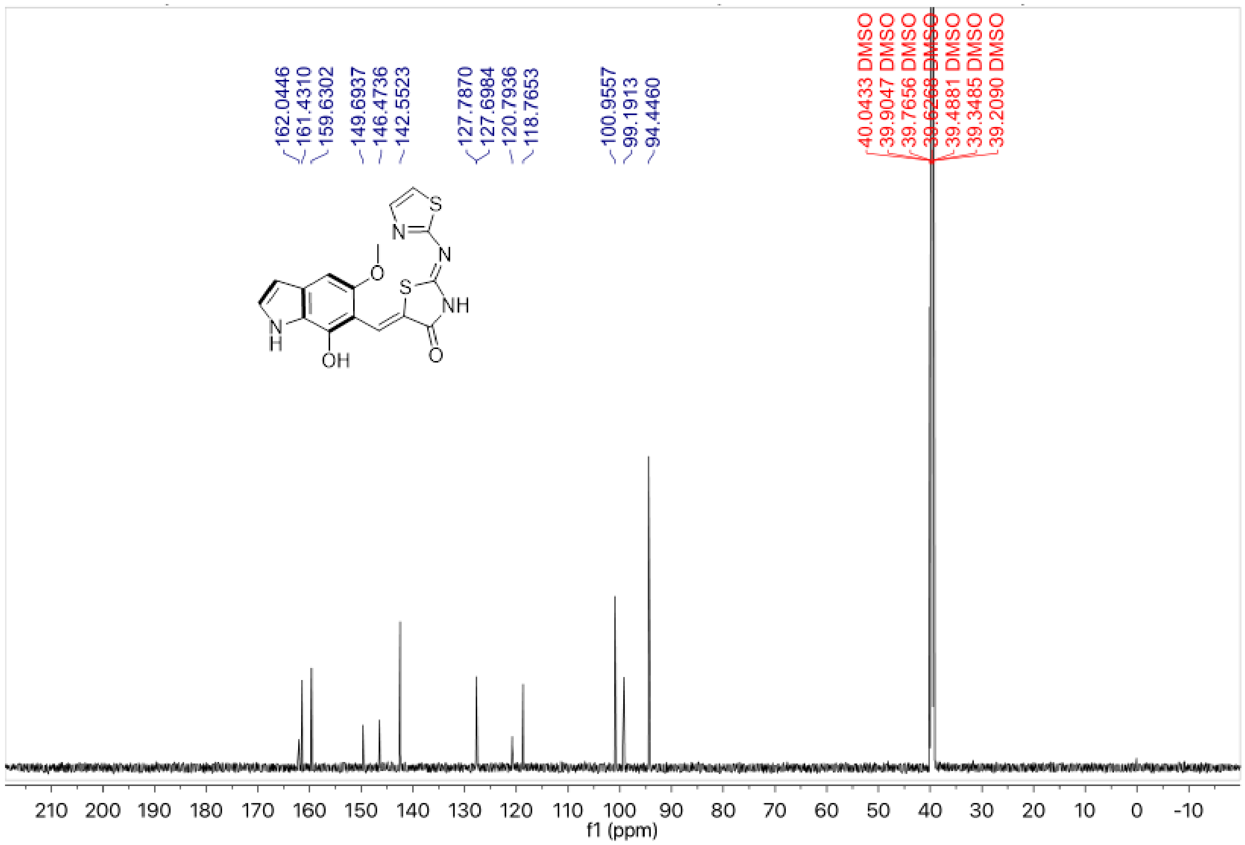

Figure S6. ^{13}C-NMR spectrum of (2Z,5Z)-5-((7-hydroxy-5-methoxy-1H-indol-6-yl)methylene)-2-(thiazol-2-ylimino)thiazolidin-4-one (8)

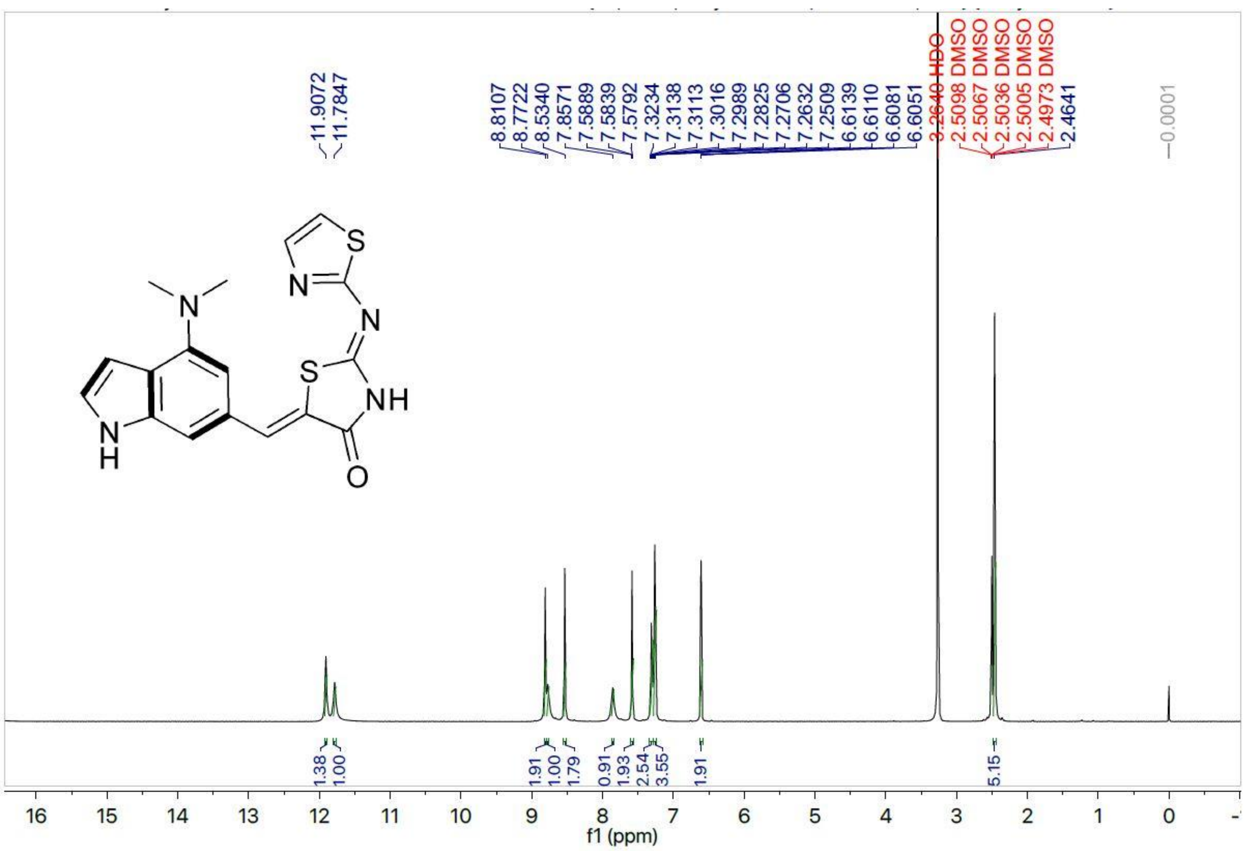

Figure S7. The proton NMR spectrum of (2Z,5Z)-5-((4-(dimethylamino)-1H-indol-6-yl)methylene)-2-(thiazol-2-ylimino)thiazolidin-4-one (9)

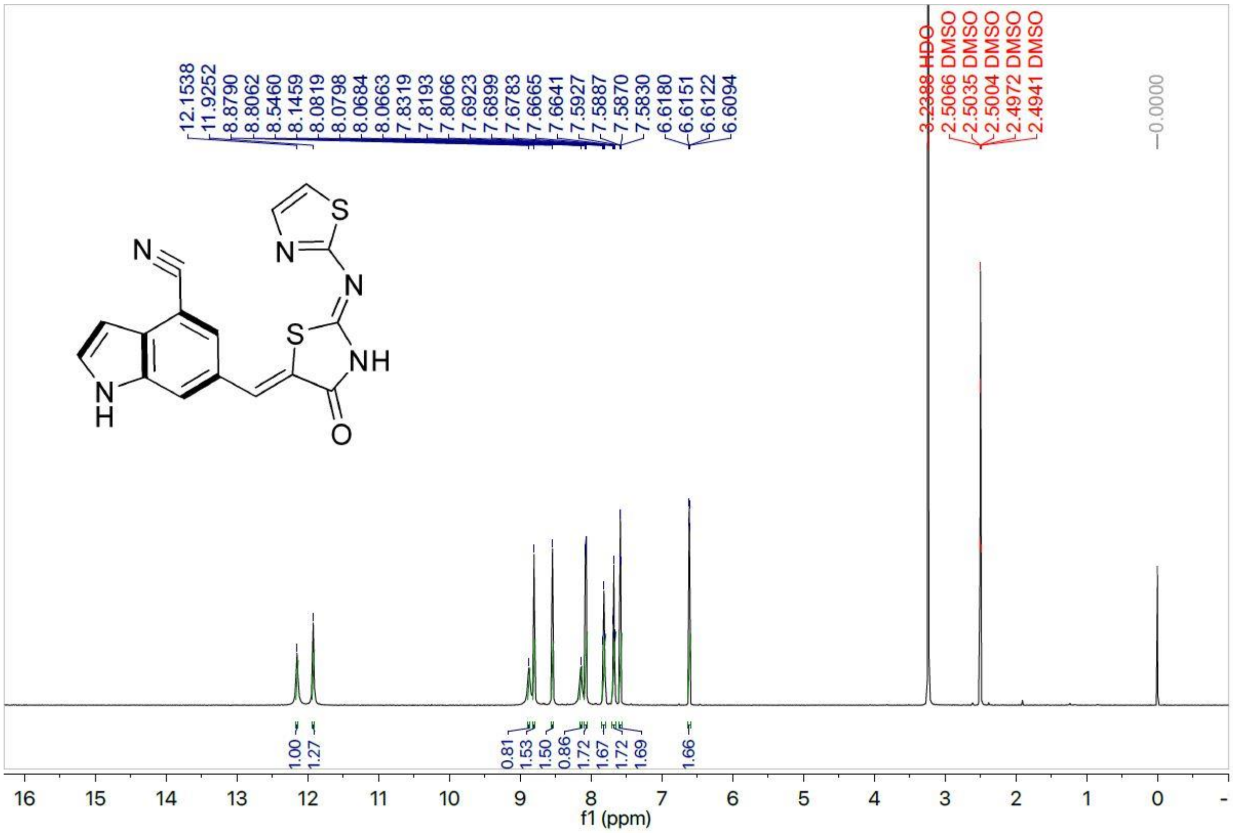

Figure S8. Proton NMR spectrum of 6-((Z)-((Z)-4-oxo-2-(thiazol-2-ylimino)thiazolidin-5-ylidene)methyl)-1H-indole-4-carbonitrile (10)

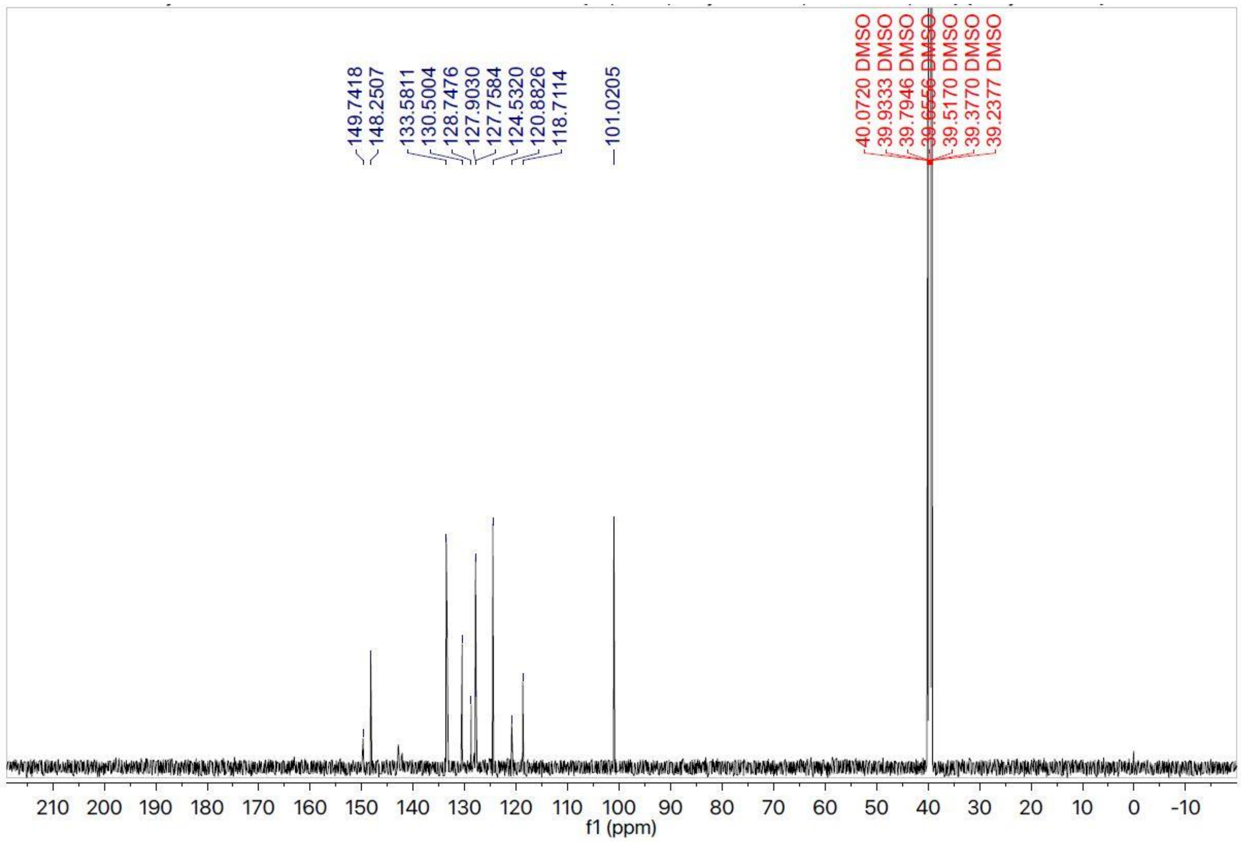

Figure S9. ^{13}C-NMR spectrum of 6-((Z)-((Z)-4-oxo-2-(thiazol-2-ylimino)thiazolidin-5-ylidene)methyl)-1H-indole-4-carbonitrile (10)

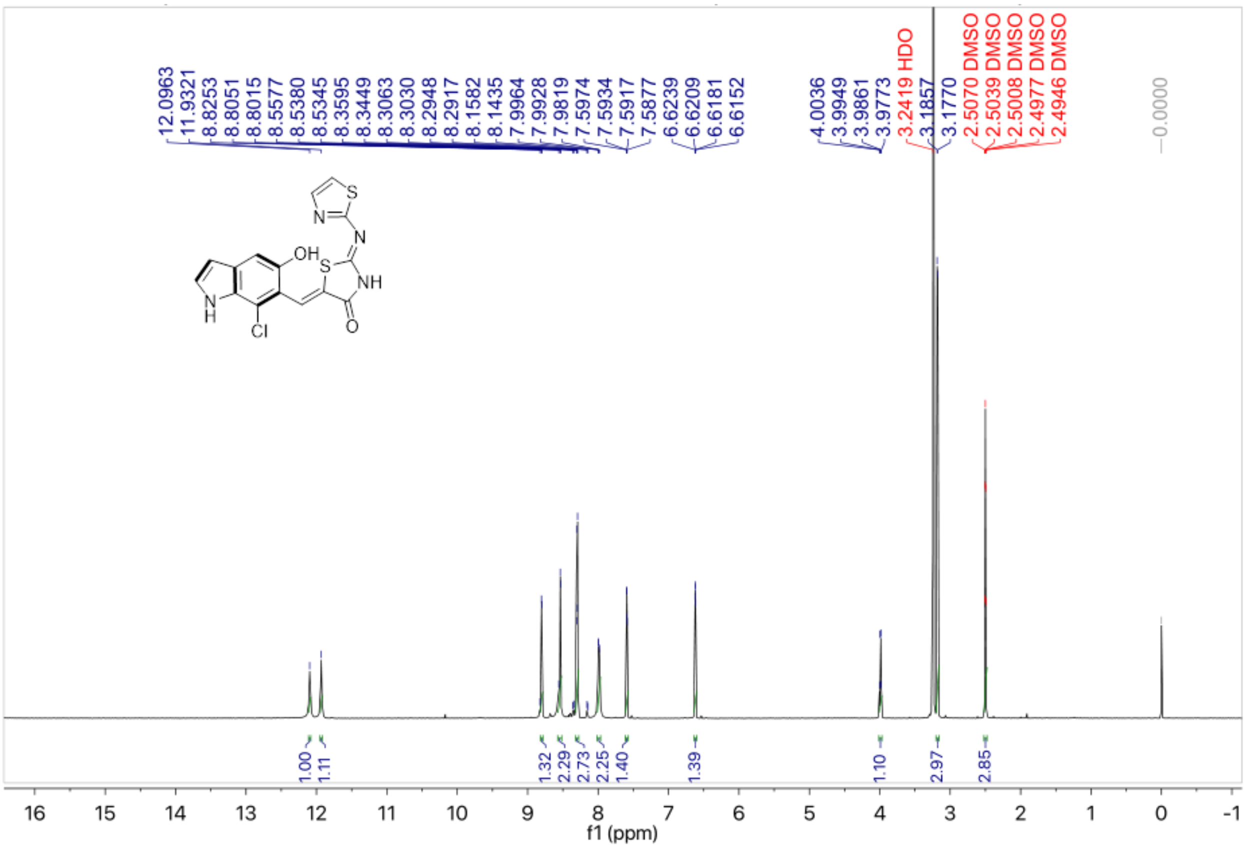

Figure S10. The proton NMR spectrum of (2Z,5Z)-5-((7-chloro-1H-indol-6-yl)methylene)-2-(thiazol-2-ylimino)thiazolidin-4-one (13)

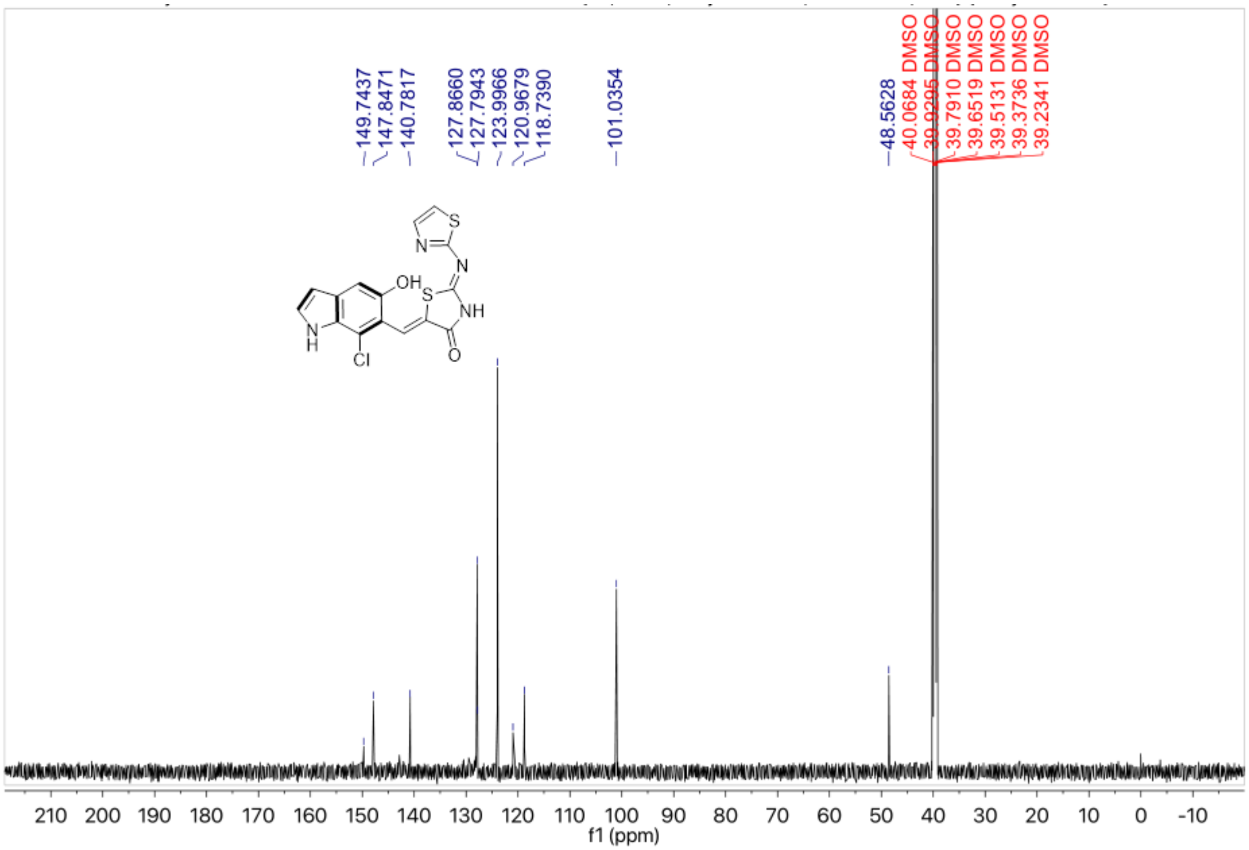

Figure S11. ^{13}C-NMR spectrum of (2Z,5Z)-5-((7-chloro-1H-indol-6-yl)methylene)-2-(thiazol-2-ylimino)thiazolidin-4-one (13)

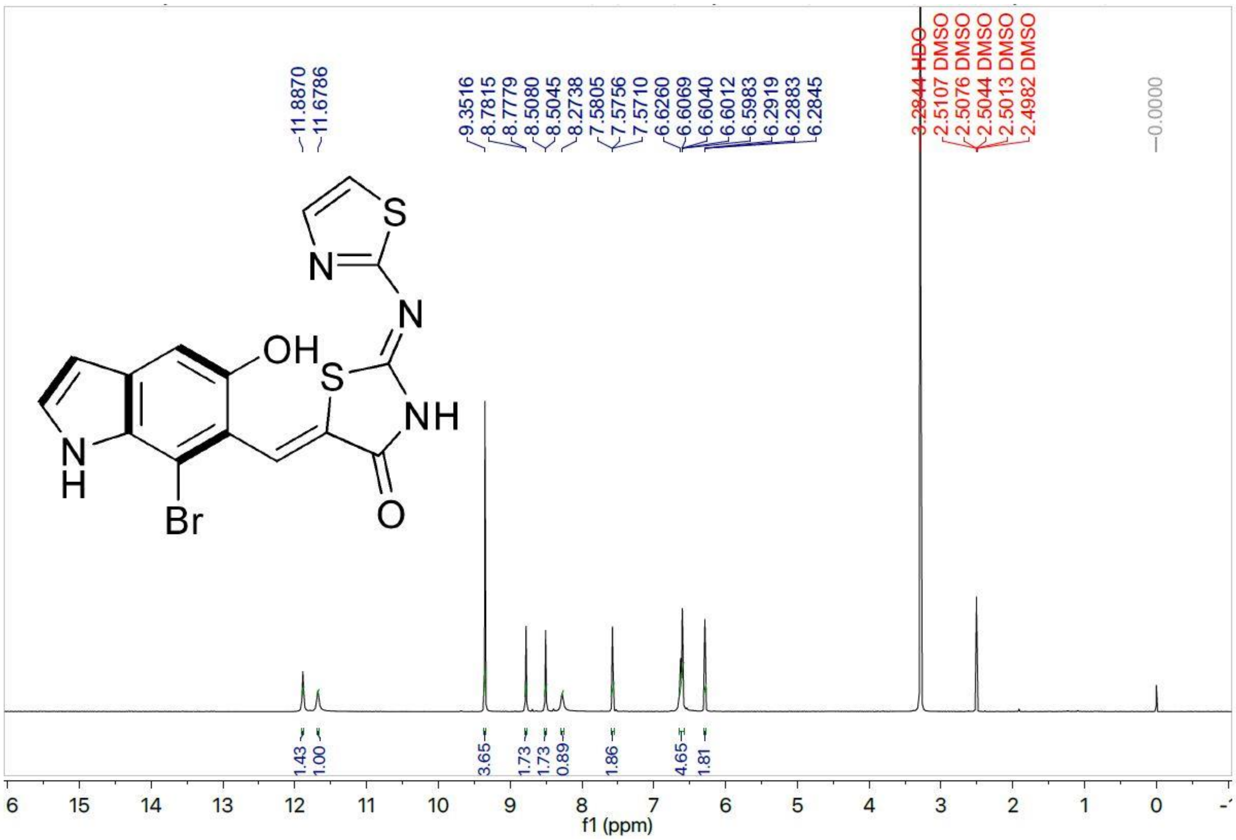

Figure S12. Proton NMR spectrum of (2Z,5Z)-5-((7-bromo-5-hydroxy-1H-indol-6-yl)methylene)-2-(thiazol-2-ylimino)thiazolidin-4-one (20)

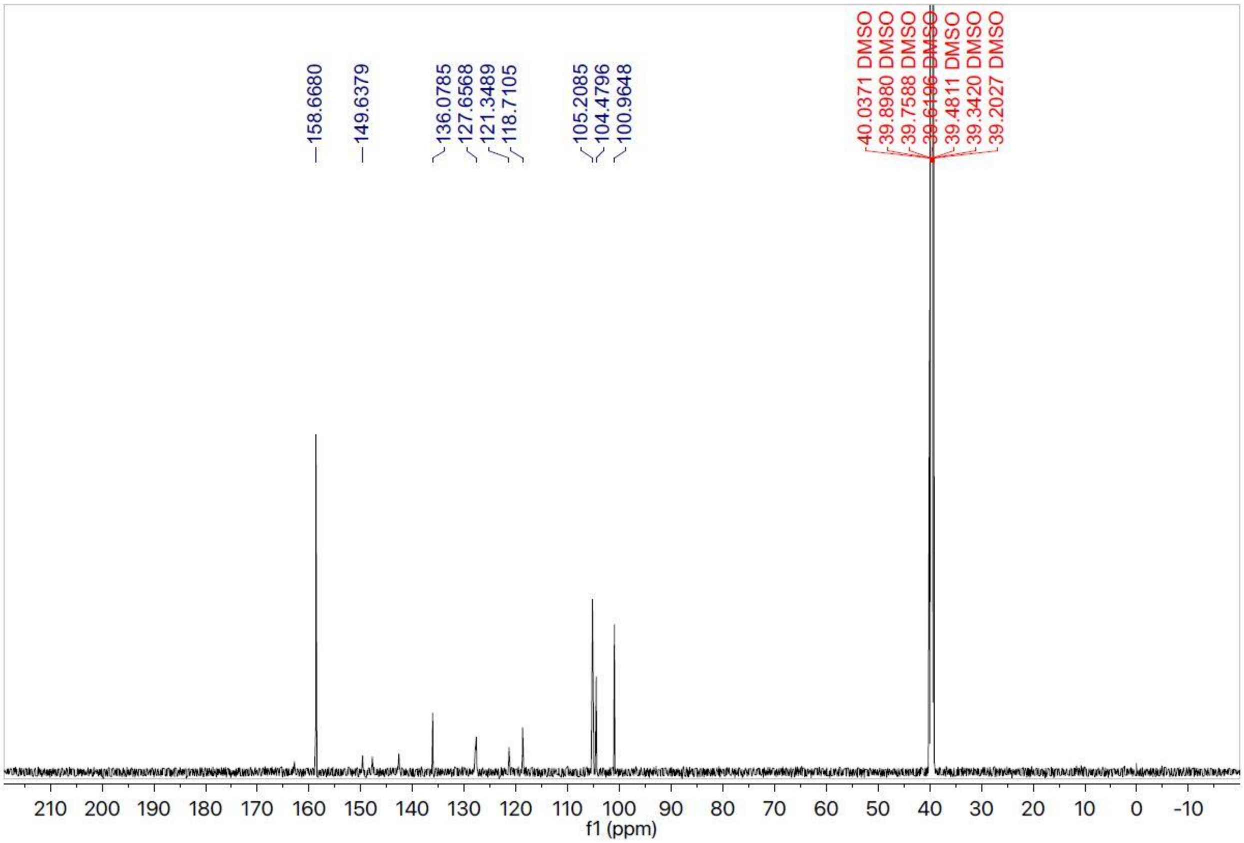

Figure S13. ^{13}C-NMR spectrum of (2Z,5Z)-5-((7-bromo-5-hydroxy-1H-indol-6-yl)methylene)-2-(thiazol-2-ylimino)thiazolidin-4-one (20)

3.4. Molecular docking protocol

The partition coefficient (log P) values were predicted using Crippen's fragmentation by the CS ChemProp module from ChemDraw Ultra 12 (CambridgeSoft, Cambridge, MA, USA) according to the fragmentation method introduced by Crippen [32]. The polar surface area (tPSA) was calculated by the atom-based method [33]. The spatial structures of compounds 4, 5, 6 and 7 were created by Chemdraw software (14.0) and transferred to auto dock tool. All atom and bond types were checked, necessary hydrogen atoms were added, and finally, the Gasteiger–Marsili charges were assigned. The pKa values for the compounds and the ionization states corresponding to the physiological conditions (pH 7.4) were assessed by Marvin (ChemAxon). The compounds were docked to alpha amylase and alpha glucosidase. Before docking with GoldSuite (CCDC), the enzyme structure was prepared. All histidine residues were protonated at Nε, the hydrogen atoms were added, and some water molecules (616, 634, 643) were retained.

The binding site was defined as all amino acid residues within the radius of approximately 12Å from the reference compound, acarbose. A standard set of genetic algorithms was applied. The population size was equal to 100, and the number of operations 100,000. As a result, 9 conformations for each compound were obtained. The results were visualized by DSV (2020). Our research group has been reported varied heterocyclic moieties also based on molecular docking[31].

3.5. Alpha-amylase inhibition protocol

Kwon and Apostolidis method were for the determination of α-amylase inhibition [34]. 500 µL (0.5 mg/mL) of α-amylase was prepared in phosphate buffer and 500 µL of sample (100, 200, 400, 800, 1000 μg/mL) were also prepared. Both solutions were incubated at 25ºC for 10 minutes. 1% Starch solution (500 µL) and 0.02 M sodium phosphate buffer was added and incubated for 10 minutes. Dinitrosalisylic acid was added as color agent, incubated in boiling water for 5 minutes, cooled and diluted using distilled water. The percentage inhibition was recorded from the absorbance using the formula

$$\%\text{inhibition} = (\text{Absorbance}_{\text{control}} - \text{Absorbance}_{\text{sample}})/\text{Absorbance}_{\text{control}} \times 100$$

3.6. Alpha-glucosidase inhibition protocol

α-Glucosidase inhibitory activities was determined as per reported methods [35]. 10 µL of test samples (5 mg/mL DMSO solution) were altered in 100 μL of 100 mM-phosphate buffer (pH 6.8) in 96-well microplate and incubated with 50 μL of crude intestinal α-glucosidase for 5 min before 50μL substrate (5 mM, *p*-nitrophenyl-α-D-glucopyranoside prepared in same buffer) was added. Release of *p*-nitrophenol was measured at 405nm spectrophotometrically (Spectra Max® plus384) for 5 min after incubation with substrate. Individual blanks for test samples were prepared to correct background absorbance where substrate was replaced with 50μL of buffer. Control sample contained 10 μL DMSO in place of test samples. Percentage of enzyme inhibition was calculated as (1-B/A) x 100 where A represents absorbance of control without test samples, and B represents absorbance in presence of test samples.

References

32 Ghose, A.K.; Crippen, G.M. Atomic physicochemical parameters for three-dimensional structure-directed quantitative structure-activity relationships. 2. Modeling dispersive and

hydrophobic interactions. J. Chem. Inf. Comput. Sci. 1987, 27, 21–35.

33 Ertl, P.; Rohde, B.; Selzer, P. Fast calculation of molecular polar surface area as a sum of fragment-based contributions and its application to the prediction of drug transport properties. J. Med. Chem. 2000, 43, 3714–3717.

34 Kharb, M.; Jat, R.K.; Parjapati, G.; Gupta, A. Introduction to molecular docking software technique in medicinal chemistry, *Int. J. Drug Res. Technol*. **2012**, 2, 189-197.

35 Zhengfu, L.; Junfeng, G.; Hongyan, Z.; Xiaoyu, Z. Adaptive molecular docking method based on information entropy genetic algorithm, *Appl. Soft Comput.***2015**, 26, 299-302.
